# Targeting the ROCK2/UBA52/DRP1 axis enhances ferroptosis and overcomes pemigatinib resistance in Cholangiocarcinoma

**DOI:** 10.1038/s41419-025-07804-9

**Published:** 2025-07-04

**Authors:** Bolin Zhang, Shan Lu, Xin Xiao, Yushu Xu, Shouhua Zhang, Leifeng Chen, Wei Zhou

**Affiliations:** 1 Department of Abdominal Surgery, Jiangxi Cancer Hospital, Nanchang, China; 2 Oncology Teaching and Research Office, The Second Affiliated Hospital of Nanchang Medical College, Nanchang, China; 3 Key Laboratory of Tumor Transformation Medicine, Jiangxi Cancer Hospital, Nanchang, China; 4 Department of General Surgery, Jiangxi Cancer Hospital, Jiangxi Medical College, Nanchang University, Nanchang, China; 5 Department of Gastroenterology and Oncology, Jiangxi Cancer Hospital, Nanchang, China; 6 Department of General Surgery, Jiangxi Children’s Hospital, Nanchang, Jiangxi Province China; 7 Department of Oncology, The Second Affiliated Hospital of Nanchang University, Nanchang, China; 8 Precision Oncology Medicine Center, The Second Affiliated Hospital of Nanchang University, Nanchang, Jiangxi Province People’s Republic of China; 9 Medical Center for Cardiovascular Diseases, Neurological Diseases and Tumors of Jiangxi Province, The Second Affiliated Hospital of Nanchang University, Nanchang, China

**Keywords:** Oncogenes, Tumour biomarkers

## Abstract

Cholangiocarcinoma (CCA) is a highly aggressive cancer that arises from the bile duct and has an extremely poor prognosis. Pemigatinib is the only Food and Drug Administration (FDA)-approved CCA-targeted drug. The CCA treatment options are insufficient considering its poor prognosis and increasing morbidity. Recently, Rho-associated coiled-coil containing protein kinase 2 (ROCK2) has been reported to promote resistance to chemotherapy. In this study, we investigated the role that ROCK2 plays in the development of resistance of CCA cells to Pemigatinib. Here, we developed Pemigatinib-resistant CCA cells, performed mRNA sequencing, retrieved The Cancer Genome Atlas (TCGA) data, and analysed ROCK2 expression in a large CCA cohort. The expression level of ROCK2 in CCA cells was significantly higher than that in adjacent noncancerous tissues. Increased expression of ROCK2 in CCA was related to a late TNM stage and decreased overall survival. Functional experiments revealed that downregulating the expression of ROCK2 promotes the ferroptosis of CCA cells, and enhances sensitivity to Pemigatinib. Moreover, upregulation of ROCK2 increased the expression of Drp1 protein. The effect of downregulating ROCK2 was reversed by Drp1 overexpression, and Drp1 knockdown inhibited Ferroptosis driven by ROCK2 overexpression. Mechanistically, ROCK2 stabilized the expression of Drp1 by competing with UBA52 to bind Drp1 and inhibiting the ubiquitination-mediated degradation of Drp1. Blocking of the UBA52– Drp1 axis inhibited the antitumour effect of Pemigatinib in ROCK2-knockdown cells both in vitro and in vivo. In conclusion, the ROCK2/UBA52/Drp1 axis is a pivotal driver of Pemigatinib resistance in CCA cells. These results provide novel insights into Pemigatinib resistance in CCA cells, suggesting that ROCK2 is a promising therapeutic target for the treatment of CCA.

## Introduction

Cholangiocarcinoma (CCA) is a highly malignant tumour that arises from epithelial cells of the bile duct mucosa and is characterised by an insidious onset and poor prognosis [1]. CCA can be classified into two main types based on the anatomical location of the tumour: intrahepatic Cholangiocarcinoma (iCCA) and extrahepatic Cholangiocarcinoma (ECC) [2]. The treatment options for CCA are limited. Surgical intervention offers the only potential cure; unfortunately, few patients are diagnosed early enough to be eligible for surgery [3]. In 2020, the United States Food and Drug Administration (FDA) approved Pemigatinib as the first targeted therapy for CCA [4]. However, Pemigatinib resistance has been reported in patients with CCA, highlighting a critical gap in our understanding of the mechanisms underlying this resistance [5–7].

Ferroptosis is a novel form of programmed cell death characterised by the accumulation of iron-dependent lipid peroxides [8]. It is often linked to an imbalance in the cellular antioxidant defense system, particularly marked by reduced levels of glutathione (GSH) and glutathione peroxidase 4 (GPX4) [9]. Cancer cells can resist ferroptosis by upregulating the expression of antioxidant enzymes, such as GPX4, and iron regulatory proteins, such as solute carrier family 7 member 11 (SLC7A11), thereby enhancing their survival [10]. Moreover, dynamin-related protein 1 (Drp1), a small guanosine triphosphatase of the dynamin family, is closely associated with tumour development and drug resistance [11]; Drp1-mediated mitochondrial fission induces cellular ferroptosis [12]. Ferroptosis regulation by Drp1 is important for understanding the mechanisms of tumour drug resistance, as drug-resistant tumour cells can evade the cytotoxic effects of chemotherapeutic agents by suppressing ferroptosis through the modulation of mitochondrial dynamics [13, 14].

Rho-associated coiled-coil containing protein kinase 2 (ROCK2) is a critical molecule in the Rho/ROCK signalling pathway [15, 16]. ROCK2 plays a significant role in the occurrence and progression of multiple tumours, exhibiting high expression levels in several cancer types, including liver, gastric, colorectal, and lung cancers [17–20]. Additionally, ROCK2 has been recognised for its role in chemoresistance. For example, the downregulation of ROCK2 expression can enhance apoptosis in colorectal cancer (CRC) cells and increase the sensitivity of CRC cells to the chemotherapeutic agent—5-fluorouracil [21]. Similarly, reduced ROCK2 levels have been linked to decreased chemotherapeutic resistance in non-small cell lung cancer cells. Meanwhile, the upregulation of ROCK2 expression has been associated with increased cisplatin resistance in bladder cancer cells [22]. In CCA cells, ROCK2 expression levels have been found to be elevated. However, the specific mechanism by which ROCK2 contributes to chemotherapeutic resistance in CCA remains unclear.

In this study, we investigated the role that ROCK2 plays in the development of Pemigatinib resistance in CCA cells. ROCK2 expression levels were found to be significantly higher in CCA tissues than that in the adjacent non-cancerous tissues, which suggested advanced disease stages and poor overall survival. Reducing ROCK2 levels promotes ferroptosis in CCA cells and enhances sensitivity to Pemigatinib by decreasing Drp1 expression. Mechanistically, ROCK2 stabilizes Drp1 by competing with UBA52, thereby preventing its degradation. This ROCK2-mediated inhibition of ferroptosis contributes to Pemigatinib resistance in CCA through the UBA52-Drp1 axis. Overall, our findings revealed that ROCK2 is a promising therapeutic target for improving treatment outcomes and overcoming drug resistance in CCA cells.

## Materials and methods

### Cell lines and reagents

The human bile duct cancer cell lines QBC-939 and RBE were supplied by the Cell Bank of Chinese Academy of Sciences (Shanghai, China). RBE cells were cultivated in Dulbecco’s Modified Eagle Medium (Gibco, Carlsbad, CA) supplemented with 100 U/mL penicillin/streptomycin and 10% fetal bovine serum (Invitrogen), whereas QBC-939 cells were cultured in Roswell Park Memorial Institute 1640 medium supplemented with 100 U/mL Penicillin/streptomycin and 10% fetal bovine serum. Both cell lines were maintained in an incubator at 37 °C with 5% CO_2_. The information of reagents and antibodies were detailed in Table S1.

### Patients and specimens

We collected 40 CCA samples and corresponding paracancerous tissues from the Department of General Surgery at Jiangxi Cancer Hospital. All cancer tissue specimens were collected from the region affected by CCA and adjacent tissues. Tissue samples were subjected to analysis using western blotting and qRT-PCR. For immunohistochemical assessments, specimens were preserved in 4% formaldehyde at room temperature. The utilization of clinical samples and associated data was ethically sanctioned by the Clinical Research Ethics and Research Committees of Jiangxi Cancer Hospital, with each patient providing informed consent.

### Tissue microarray and IHC

Tissue microarray (TMA) was provided by Qilu Hospital. For Immunohistochemistry, paraffin-embedded tissue sections were deparaffinized, and antigen retrieval was achieved in 10 mmol/L sodium citrate buffer (pH 6.0). The tissue section was incubated with primary antibodies of ROCK2 (1:200), Drp1 (1:200) at 4 °C overnight. The appropriate secondary antibodies (Zenbio, China) were applied for 30 min at room temperature. Subsequently, the slides were incubated with conjugated horseradish peroxidase streptavidin. The peroxidase reaction was developed using a 3,3-diaminobenzidine (DAB) solution (MCE).

### RNA extraction and qRT-PCR

Total RNA was extracted from cultivated CCA cells using TRIZOL reagent(Invitrogen, 15596026), quantified on the Evolution 350 ultraviolet–visiblespectrophotometer (Thermo), reverse-transcribed using the PrimeScript kit with gDNA Eraser (Takara, RR047A), and subjected to qRT-PCR using the TB Green® PreMix Ex Taq Quantity (Tli RNaseH Plus) kit (Takara, RR420A). The expression levels of ROCK2 and Drp1 were determined using the 2-ΔΔCt method and normalized to their respective controls. The controls used were β-actin(for ROCK2 and Drp1). All measurements were performed in triplicate. Primers were acquired from Genomeditech (Guangzhou, China), and their sequences are given in Table S2.

### Western blotting

Total protein was extracted from CCA cells using radioimmunoprecipitation assay lysis buffer, quantified using the bicinchoninic acid assay, mixed with protein loading buffer, and boiled for 10 min. Proteins were resolved by electrophoresis on 7.5% or 10% sodium dodecyl sulfate–polyacrylamide gels and transferred to a 0.22-μm polyvinylidene difluoride membrane. The membrane was blocked with 5% skim milk for 60 min at room temperature (RT), then incubated with the appropriate primary antibodies at 4 °C overnight. After washing three times for 10 min each with 1× Tris-buffered saline with Tween 20, the membrane was incubated with the appropriate secondary antibodies for 1 h at RT, washed again three times for 10 min each with 1× Tris-buffered saline with Tween 20, and exposed to enhanced chemiluminescence reagents for imaging.

### Cell transfection

All plasmids were purchased from Genechem. The information of plasmids and primer sequences are describe in Tables S3 and S4.

### Cell viability assay

CCA cells in their logarithmic growth phase were seeded in a 96-well plate (3 × 10^3^ cells/100 μL) and incubated for 1–4 days. Every 24 h, 10 μl of CCK-8 reagent (10 μL/100 μL; GLPBIo, GK10001) was added and incubated at 37 °C for 2 h. The absorbance value at 450 nm was detected using a spectrophotometer.

### Colony formation assay

CCA cells were seeded in a 6-well plate (1500 cells/well), cultured for 2 weeks in a humidified atmosphere with 5% CO_2_, stained for 15 min with 0.1% crystal violet, and washed twice with phosphate-buffered saline (PBS). Allow the wells to air dry, and then visualize and count the colonies.

### 5-ethynyl-2’-deoxyuridine (EdU) assay

Cell proliferation was determined using an EdU Proliferation Kit (Beyotime, C0071S, Shanghai, China). Cells were cultured in a 48-well plate for 24 h, then incubated with 50 mM EdU solution for 2 h and fixed in 4% paraformaldehyde. Subsequently, the cells were permeabilized with 0.25%Triton X-100 for 15 min and sequentially stained with Azide 488 and Hoechst. Finally examined under a fluorescence microscope.

### Immunofluorescence

CCA cells that had undergone differentiation were seeded in 24-well plates (1.5 × 10^4^ cells/mL), cultured for 1 day, fixed for 15 min with 4% paraformaldehyde, washed three times with PBS for 3 min each, permeabilized with 0.5% Triton X-100 at RT for 20 min. After another three washes with PBS, the cells were blocked with 500 μL/well of 5% goat serum incubated at RT for 30 min to prevent nonspecific binding. The cells were then incubated overnight at 4 °C with antibodies against ROCK2, Drp1, UBA52, washed three times with PBS containing Tween 20, incubated with fluorescent secondary antibodies for 1 h, sequentially stained with Hoechst (Beyotime, 33342, Shanghai, China) to visualize the nuclei, washed three more times with PBS containing Tween 20, Photographs were taken using a confocal microscope.

### Biochemical assay

Commercially available assay kits measured MDA (Beyotime, S0131S, Shanghai, China) and GSH/GSSG(Beyotime, S0053, Shanghai, China) concentrations or activity in specific samples, following the manufacturer’s guidelines.

### ROS and Fe^2+^ imaging

Cells were planted on 6-well chamber slides (5 × 10^5^ cells/well) for 24 h. The slides were washed with PBS and incubated with PBS containing 2 mM DCFH-DA(Beyotime, S0033S, Shanghai, China) or 1 μM FerroOrange (DojinDo, F374, Japan) for 20 min. sequentially stained with Hoechst (Beyotime, 33342, Shanghai, China) to visualize the nuclei. Finally examined under a fluorescence microscope.

### Mass spectrometry

Liquid chromatography (LC) with tandem mass spectrometry (MS) was carried out by PTMBIO (Hangzhou, Jiangsu,China). Protein pellets were digested with trypsin to a protein ratio of 1:20 and incubated at 37 °C for 4 h. For each sample, the equivalent of 2–5 mg of protein was loaded into the LC-MS/MS.

### Subcutaneous xenograft experiments

CCA cells (1 × 10^6^ in 100 ml PBS) lentivirally transduced with firefly luciferase (Fluc) were injected subcutaneously into the flanks of nude mice (male BALB/c-nu/nu, 6–8 weeks old). The IVIS Imaging System was used to measure tumor growth. The tumor volume (*V*) was calculated as follows: *V* = 0.52 × length × width^2^. Experimental nude mice were euthanized at the end of the observation period, and then the tumors were removed and imaged. All animal studies were approved by the Animal Experimental Ethics Committee of Nanchang University and were performed in accordance with the NIH Guide for the Care and Use of Experimental Animals.

### GST pull-down assays

Purified protein (GST, GST-UBA52, His-ROCK2, His-Drp1) purchased from AtaGenix(Wuhan, Hubei, China), Glutathione Sepharose 4B beads (sigma) were incubated with GST, GST–UBA52 along with purified His-ROCK2 or His-Drp1 in incubation buffer (20 mmol/L Tris-HCl, pH 7.4, 0.1% Triton X-100) overnight at 4 °C and washed five times with incubation buffer. Proteins were eluted by boiling at 95 °C for 5 min with SDS buffer, resolved by SDS-PAGE, and subjected to western blot analysis.

### Co-immunoprecipitation

Protein sample preparation was the same as western blotting before the addition of loading buffer. For UBA52 and ROCK2/Drp1, ROCK2 and Drp1 interaction, every 1 mg protein sample was incubated with 2 μg primary antibody at 4 °C overnight. Protein A/G beads (MCE) were added and incubated at 4 °C for 2 h. Then, sediments were collected after centrifuge at 14,000 rpm at 4 °C for 1 min and washed three times with RIPA lysis buffer. Protein A/G beads were washed with 1× loading buffer to remove unbound proteins. Precipitated proteins were analyzed by immunoblotting.

### Cell knockout

The UBA52 knockout Cholangiocarcinoma cell line was constructed by Nanchang Focus Biology Co., Ltd.

### Statistical analysis

Bioinformatic statistical analysis was performed using Rv4.1.2 (https://www.r-project.org/). The results are expressed as the mean ± standard deviation of three independent experiments. The Fisher precision test evaluated the relationship between clinicopathological features and ROCK2 and Drp1 expression in patients with Cholangiocarcinoma. For comparisons between two groups, we used the Student’s *t*-test when the data were normally distributed. If the data did not meet the assumption of normality, we used the Mann-Whitney U test as a non-parametric alternative. For comparisons involving three or more groups, we applied one-way ANOVA to assess differences among the groups. SPSS 21.0 (IBM Corp.) and GraphPad Prism 8.0 (GraphPad Software, Inc.) were used for statistical analysis and construction of all graphs, respectively. *p* < 0.05 was considered statistically significant.

## Results

### ROCK2 is strongly expressed in Pemigatinib-resistant Cholangiocarcinoma cells and leads to poor prognosis

**Fig. 1:**
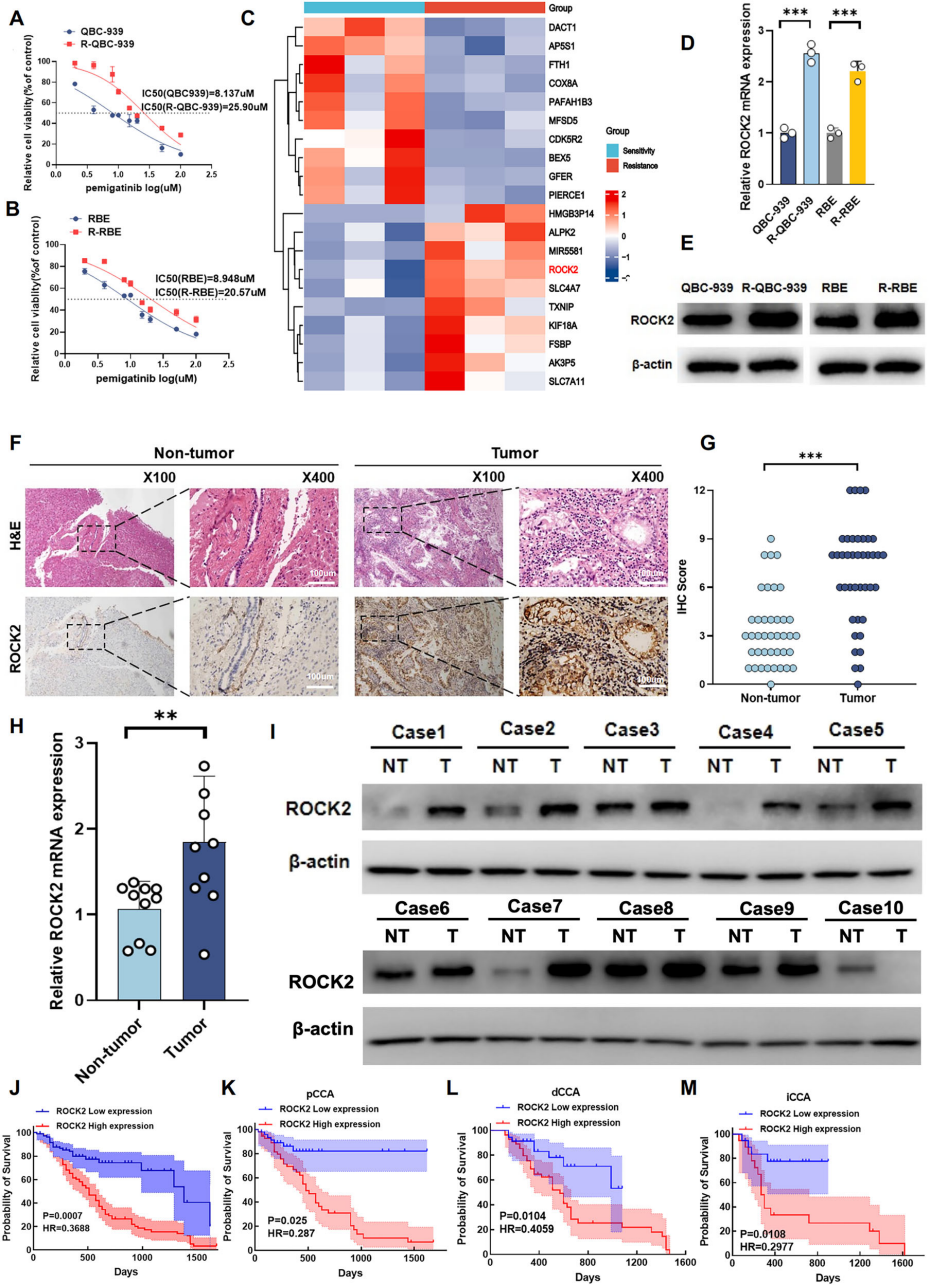
ROCK2 is strongly expressed in pemigatinib-resistant CCA cells and plays a key role in the poor prognosis of patients with CCA. **A**, **B** The IC50 values of Pemigatinib in QBC-939, R-QBC-939, RBE, and R-RBE cells were measured using cell viability assays. Inhibition curves were fitted by nonlinear regression, and IC50s were calculated using GraphPad Prism 9 software. **C** RNA-seq analysis showed differentially expressed genes in QBC-939 and R-QBC939 cells. **D**, **E** ROCK2 expression was detected by western blot and qPCR in pemigatinib-resistant and parental cell lines (*n* = 3). Data are presented as mean ± SD. ***P < 0.001. **F**, **G** Representative immunohistochemical staining of ROCK2 in CCA tissues and their adjacent tissues (*n* = 40, Scale bar: 100 μm). Data indicated as mean ± SD. ***P < 0.001. **H**, **I** Representative western blot and qRT-PCR analyses were performed to assess ROCK2 expression in tumour (*n* = 10) and adjacent normal (*n* = 10) CCA tissues. Data indicated as mean ± SD. **P < 0.01. **J**–**M** The correlation between ROCK2 expression and overall survival in patients with CCA (*n* = 233, *n* (low expression) = 109, *n* (high expression) = 124), pCCA (*n* = 105, *n* (low expression) = 49, *n* (high expression) = 56), dCCA (*n* = 90, *n* (low expression = 35), *n* (high expression) = 55), and iCCA (*n* = 38, *n* (low expression = 19), *n* (high expression) = 19). Low expression:Remmele score ≤ 6, high expression:Remmele score > 6. The statistical significance was analyzed with log-rank test. The accuracy of the prognostic value of different expression patterns was evaluated with HR (hazard ratio).

To characterise Pemigatinib resistance in CCA, we established two Pemigatinib-resistant CCA cell lines (RBE-R and QBC-939-R) through chronic exposure of the cells to progressively increasing concentrations of Pemigatinib. Compared to that shown by their parental cell lines (RBE and QBC-939), the RBE-R and QBC-939-R cells exhibited reduced sensitivity to Pemigatinib, as indicated by high IC50 values, enhanced colony formation ability, and increased levels of 5-ethynyl-2′-deoxyuridine (EdU)-positive cells upon Pemigatinib treatment of the Pemigatinib-resistant cells (Figs. 1A, B and S1A, B). Next, to identify the key genes associated with Pemigatinib resistance in CCA, we conducted RNA sequencing (RNA-seq) analysis using RBE and RBE-R cell samples. This analysis revealed 1443 differentially expressed genes, of which ROCK2 was significantly upregulated in Pemigatinib-resistant CCA cells (Fig. 1C and S1C). Furthermore, the results of western blotting and real-time quantitative reverse transcription PCR (qRT-PCR) analyses revealed ROCK2 as highly expressed in Pemigatinib-resistant CCA cells (Fig. 1D, E). These findings suggested that ROCK2 plays a critical role in the development of Pemigatinib resistance in CCA cells.

Next, we examined ROCK2 expressions in patients with CCA. Immunohistochemical (IHC) analysis results indicated that, compared to that in the adjacent non-tumour tissues, the frequency of ROCK2-positive cells was higher and the staining intensity was stronger in CCA tissues from patients who showed a poor response to Pemigatinib (Fig. 1F, G). The results of qRT-PCR and western blot analyses confirmed a significant increase in ROCK2 expression levels in CCA tissues compared to that in adjacent non-tumour tissues (Fig. 1H, I). ROCK2 was highly expressed in CCA cells according to The Cancer Genome Atlas (TCGA) database (Fig. S1D). Furthermore, we analysed ROCK2 expression levels in all CCA subtypes and categorised the patient cohort into subsets with low or high ROCK2 expression levels (Tables S5). Notably, high ROCK2 expression levels were associated with poor prognoses in patients with iCCA, perihilar Cholangiocarcinoma (pCCA), and distal Cholangiocarcinoma (dCCA) (Fig. 1J–M). Collectively, these data suggested that ROCK2 may play a significant role in Pemigatinib resistance, underscoring its potential as a biomarker for CCA prognosis and as a therapeutic target.

### ROCK2 inhibits ferroptosis and leads to Pemigatinib resistance in Cholangiocarcinoma cells

**Fig. 2:**
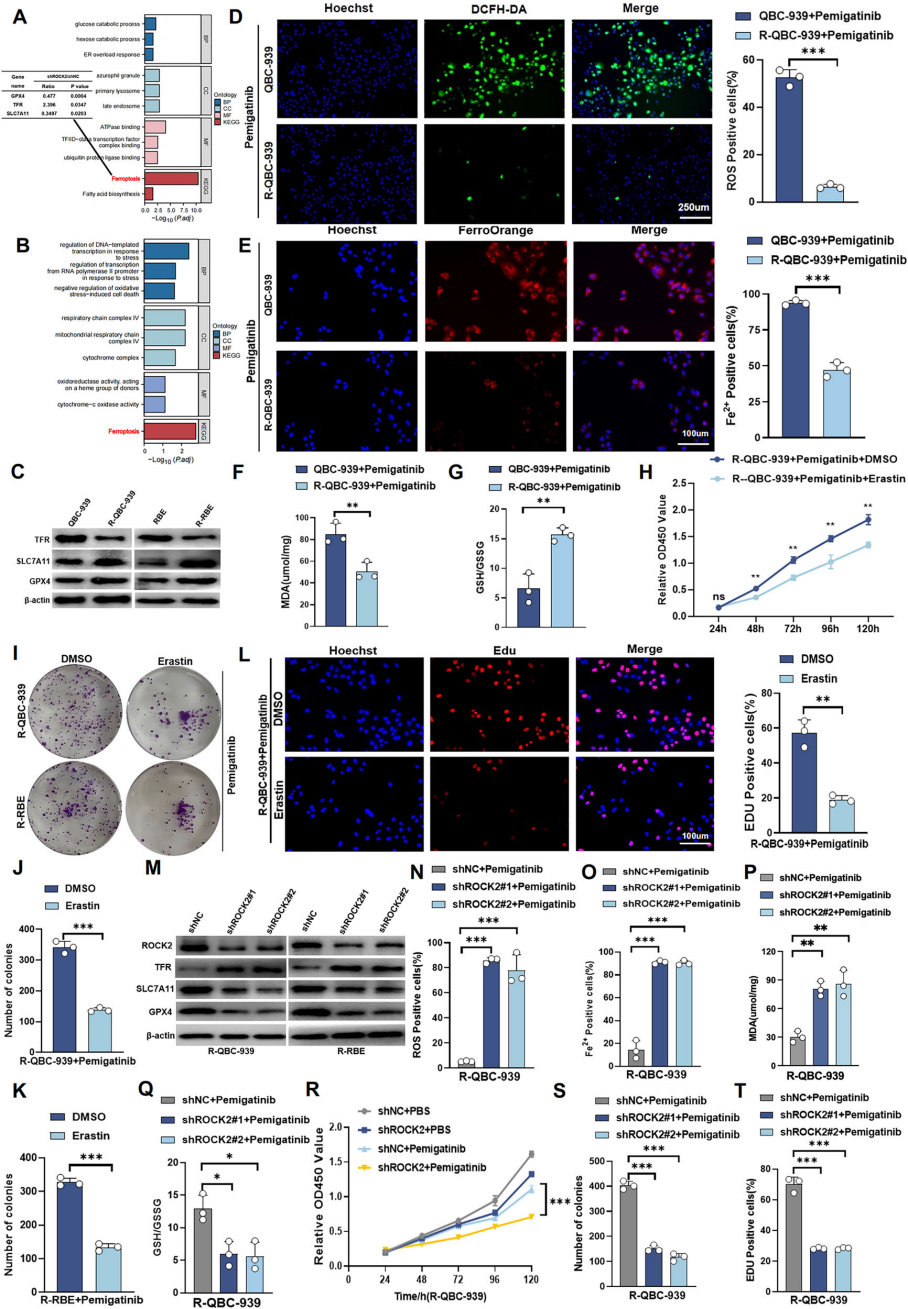
ROCK2 inhibits ferroptosis to regulate pemigatinib resistance in Cholangiocarcinoma cells. **A** Proteomics sequencing was performed using control and ROCK2 knockdown R-QBC-939 cells. KEGG (Kyoto Encyclopedia of Genes and Genomes) pathway enrichment analysis revealed significant enrichment of ferroptosis-related pathways. **B** RNA-seq was performed using QBC-939 and R-QBC-939 cells.KEGG pathway enrichment analysis revealed significant enrichment of ferroptosis-related pathways. **C** The expression of SLC7A11, GPX4, and TFR was detected by western blot in RBE, R-RBE, QBC-939, and R-QBC-939 cells. **D** DCFH-DA (detection probe)was used to determine the content of Reactive Oxygen Species (ROS) in QBC-939 and R-QBC-939 cells after treatment with Pemigatinib(5 μM, 48 h at 37 °C). Scale bar = 250 μm. Data indicated as mean ± SD.***P < 0.001. **E** FerroOrange (detection probe) was used to determine the content of ferrous ions in QBC-939 and R-QBC-939 cells after treatment with Pemigatinib(5 μM, 48 h at 37 °C). Data indicated as mean ± SD. Scale bar = 100 μm. ***P < 0.001. **F** MDA assay kit was used to determine the MDA content in QBC-939 and R-QBC-939 cells after treatment with Pemigatinib (5 μM, 48 h at 37 °C). Data indicated as mean ± SD. **P < 0.01. **G** GSH/GSSG assay kit was used to determine the GSH/GSSG ratio in QBC-939 and R-QBC-939 cells after treatment with Pemigatinib (5 μM,48 h at 37 °C). Data indicated as mean ± SD. **P < 0.01. **H** Cell viability was assessed in R-QBC-939 cells after treatment with Pemigatinib (5 μM, 48 h at 37 °C) and Erastin (2 μM, 48 h at 37 °C) using CCK-8 assay. Data indicated as mean ± SD. **P < 0.01, ns, nonsignificant. **I**–**L** Cell proliferation was measured using clone formation and EdU assays in R-QBC-939 and R-RBE cells after treatment with Pemigatinib (5 μM, 48 h at 37 °C) and Erastin (2 μM, 48 h at 37 °C). Data indicated as mean ± SD. **P < 0.01, ***P < 0.001. **M** The expression of SLC7A11, GPX4, and TFR was detected by Western blot in ROCK2-knockdown R-QBC-939 and R-RBE cells. **N**–**Q** Intracellular ROS levels, ferrous ion activity, MDA content, and GSH/GSSG ratio were measured in control and ROCK2 knockdown R-QBC-939 cells after treatment with Pemigatinib (5 μM, 48 h at 37 °C). Data indicated as mean ± SD. ***P < 0.001,**P < 0.01,*P < 0.05. **R**–**T** Cell proliferation was measured using CCK-8 assay, clone formation assay, and EdU assay in control and ROCK2-knockdown R-QBC-939 cells after treatment with Pemigatinib (5 μM, 48 h at 37 °C). Data indicated as mean ± SD.***P < 0.001.

We performed a comprehensive analysis to investigate the role of ROCK2 in Pemigatinib resistance in CCA cells. Using Kyoto Encyclopedia of Genes and Genomes(KEGG) and Gene Ontology (GO) analysis, we discovered that ROCK2 is involved in the regulation of ferroptosis in CCA cells(Fig. 2A). RNA-seq analysis of RBE and RBE-R cell samples indicated a strong correlation between ferroptosis and Pemigatinib resistance in CCA cells (Fig. 2B). In addition, biliary duct cancer cells, which were exposed to Pemigatinib, were treated with the iron-mediated death inhibitor deferoxamine (DFO), apoptosis inhibitor—Z-VAD-FMK, necrostatin-1, and autophagy inhibitor 3-methyladenine. DFO significantly inhibited the effect of Pemigatinib on Cholangiocarcinoma (Fig. S2A). Based on these findings, we hypothesised that ROCK2 leads to Pemigatinib resistance in CCA cells because it is involved in ferroptosis. To test this hypothesis, we explored the relationship between ferroptosis and the sensitivity of CCA cells to Pemigatinib. We determined the expression levels of transferrin receptor (TFR), SLC7A11, and GPX4 in RBE, RBE-R, QBC-939, and QBC-939-R cells using western blot analysis. SLC7A11 and GPX4 were highly expressed and TFR was expressed at low levels in RBE-R and QBC-939-R cells (Fig. 2C). The analysis of reactive oxygen species (ROS), Fe^2+^, malondialdehyde (MDA), and GSH/glutathione disulphide (GSSG) levels in parental and Pemigatinib-resistant CCA cells revealed that ferroptosis was significantly inhibited in Pemigatinib-resistant CCA cells following Pemigatinib treatment (Figs. 2D–G and S2B–E). Furthermore, we investigated the effects of the ferroptosis inducer, erastin, on Pemigatinib-resistant CCA cells. We conducted a series of experiments including cell counting kit-8 (CCK-8), colony formation, and EdU assays; the results revealed that treatment with erastin significantly enhanced the sensitivity of Pemigatinib-resistant CCA cells to Pemigatinib (Figs. 2H–L and S2F–H). These findings suggested that erastin not only induces ferroptosis but also plays a crucial role in reversing Pemigatinib resistance in CCA cells.

Next, we investigated the effects of ROCK2 knockdown in RBE-R and QBC-939-R cells on ferroptosis-related protein expression levels using western blot analysis. ROCK2 knockdown increased TFR expression levels and significantly reduced GPX4 and SLC7A11 levels (Fig. 2M). To further evaluate the effect of ROCK2-knockdown on ferroptosis, we measured ROS, Fe^2+^, MDA, and reduced GSH/GSSG levels in shNC-Pemigatinib-resistant and shROCK2-Pemigatinib-resistant CCA cells. Ferroptosis was significantly elevated in shROCK2 Pemigatinib-resistant CCA cells following Pemigatinib treatment (Figs. 2N–Q, S2I, and S3A–G). These results suggested that the downregulation of ROCK2 enhanced ferroptosis in Pemigatinib-resistant CCA cells. ROCK2-knockdown in cells significantly enhanced the sensitivity of these cells to Pemigatinib resistant, and our results also showed that while inhibition of ferroptosis with FDO significantly reduced the cell death induced by ROCK2 knockdown, none of the other inhibitors (Z-VAD, Ne, or 3-MA) had a comparable effect in CCA cells (Figs. 2R–T, S4A–G, S5A–G). Taken together, these findings suggested that ROCK2 plays a critical role in inhibiting ferroptosis, which in turn affects the resistance of CCA cells to Pemigatinib.

### ROCK2 induces Pemigatinib resistance in Cholangiocarcinoma cells by inhibiting ferroptosis in vivo and in vitro

**Fig. 3:**
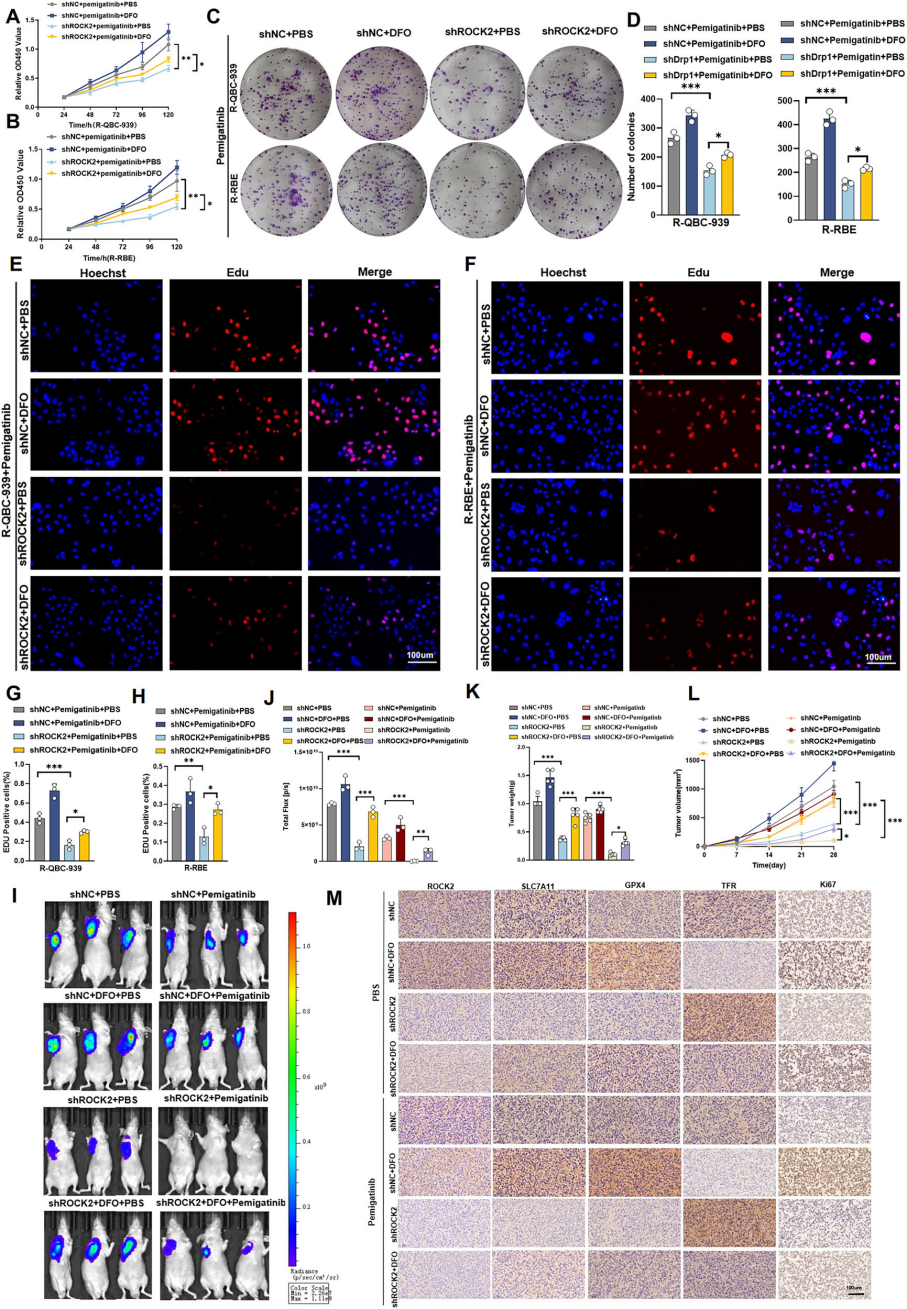
ROCK2 induces pemigatinib resistance in Cholangiocarcinoma cells by inhibiting ferroptosis. **A**–**H** Cell proliferation was measured using CCK-8 assay, clone formation assay, and EdU assay in control and ROCK2-knockdown R-QBC-939 cells after treatment with Pemigatinib(5 μM, 48 h at 37 °C) and Deferoxamine (10 μM, 48 h at 37 °C). Data indicated as mean ± SD. ***P < 0.001,**P < 0.01, *P < 0.05. **I**, **J** In vivo subcutaneous xenografts were established using stable ROCK2-silenced QBC-939 cells in nude mice, followed by treatment with Pemigatinib (10 mg/kg) or Deferoxamine (100 mg/kg). Bioluminescence imaging was performed at 28 days post-inoculation (*n* = 3). Data indicated as mean ± SD. ***P < 0.001, **P < 0.01. **K**, **L** Tumour weight was measured at the end of the experiment, while tumour volume was monitored every 7 days, allowing the corresponding tumour growth curves to be plotted (*n* = 5). Data indicated as mean ± SD. ***P < 0.001, *P < 0.05. **M** Representative IHC staining of ROCK2, GPX4, TFR, SLC7A11, and Ki67 in allograft tumor tissues (*n* = 5). Scale bar: 100 μm.

To further confirm that ROCK2 leads to Pemigatinib resistance in CCA cells through ferroptosis, ROCK2-knockdown drug-resistant CCA cells were administered the ferroptosis inhibitor DFO. CCK-8 assay results revealed that the decrease in ROCK2 levels enhanced the sensitivity of drug-resistant CCA cells to Pemigatinib; however, the addition of the ferroptosis inhibitor DFO blocked this process (Fig. 3A, B). We performed colony formation and EdU assays to validate these results. Colony formation assay results demonstrated that the decrease in ROCK2 levels significantly decreased the number of colonies formed by Pemigatinib-treated drug-resistant CCA cells; addition of the ferroptosis inhibitor DFO restored the colony formation ability of these cells (Fig. 3C, D). The EdU assay results corroborated these findings; ROCK2-knockdown lead to a decrease in Pemigatinib-treated drug-resistant CCA cell proliferation; the addition of DFO restored the proliferative ability of these cells (Fig. 3E–H). Thus, our findings demonstrated that ROCK2 mediates Pemigatinib resistance in CCA cells by regulating ferroptosis in vitro. Subsequently, we conducted a nude mouse xenograft assay to assess whether ROCK2 leads to Pemigatinib resistance in CCA cells by regulating ferroptosis in vivo. We found that ROCK2-knockdown increased the sensitivity of CCA cells to Pemigatinib, while the addition of DFO blocked this process, and there is no significant difference in the total weights of the mice (Fig. 3I–L and S6A, B). Additionally, IHC analysis revealed that TFR levels were increased and Ki67, GPX4, and SLC7A11 levels were decreased in shROCK2-R-QBC-939 tumours. Notably, the addition of DFO blocked this process (Fig. 3M). Based on these results, we concluded that ROCK2 induced Pemigatinib resistance in CCA cells, primarily by inhibiting ferroptosis, both in vivo and in vitro.

### ROCK2 regulates Drp1 expression and ROCK2 and Drp1 expression levels are positively correlated in CCA tissues

**Fig. 4:**
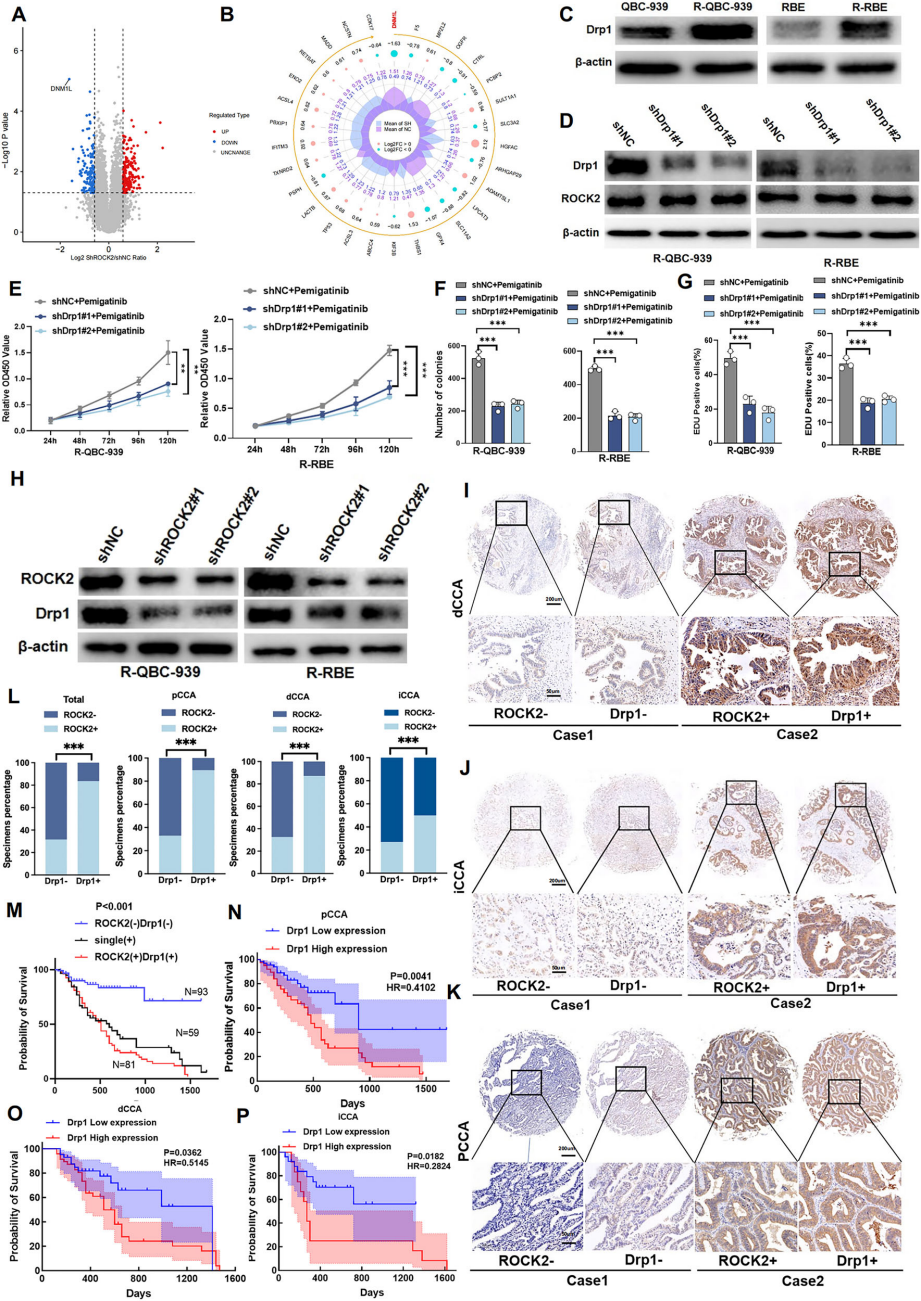
ROCK2 regulates dynamin-related protein 1 expression and ROCK2 and Drp1 expression are positively correlated in CCA tissues. **A**, **B** Proteomics sequencing analysis was performed to identify differentially expressed proteins in control and ROCK2-knockdown R-QBC-939 cells. **C** The expression of Drp1 was detected by Western blot in RBE, R-RBE, QBC-939, and R-QBC-939 cells. **D** The expression of Drp1 and ROCK2 was detected by western blot in control and Drp1-knockdown R-QBC-939 and R-RBE cells. **E**–**G** Cell proliferation was measured using CCK-8 assay, clone formation assay, and EdU assay in control and Drp1-knockdown R-QBC-939 and R-RBE cells after treatment with Pemigatinib (5 μM, 48 h at 37 °C). Data indicated as mean ± SD. ***P < 0.001, **P < 0.01. **H** The expression of Drp1 and ROCK2 was detected by western blot in control and ROCK2-knockdown R-QBC-939 and R-RBE cells. **I**–**K** Representative IHC staining of ROCK2 and Drp1 in pCCA, dCCA and iCCA. (top: ×100 magnification, scale bar, 200 μm; bottom: ×400 magnification, scale bar, 50 μm). **L** Patients were divided into subsets with low or high Drp1 expression according to the Remmele score. The ROCK2 Remmele score was compared between the low and high Drp1 subsets. Low expression:Remmele score ≤ 6, high expression:Remmele score > 6. Statistical significance was analyzed with chi-square test. ***P < 0.001. **M** Patients with different expression patterns of ROCK2 and Drp1 had different prognoses. The statistical significance was analyzed with log-rank test. The accuracy of the prognostic value of different expression patterns was evaluated with HR. **N**–**P** The correlation between the Drp1 expression and overall survival in patients with pCCA (*n* = 105, *n* (low expression) = 67, *n* (high expression)=38), dCCA (*n* = 90, *n* (low expression = 43), *n* (high expression) = 47), and iCCA(*n* = 38, *n* (low expression=26),n(high expression)=12)). Low expression:Remmele score ≤ 6, high expression:Remmele score > 6. The statistical significance was analyzed with log-rank test. The accuracy of the prognostic value of different expression patterns was evaluated with HR.

To further investigate the mechanism by which ROCK2 modulates ferroptosis in CCA cells, we conducted a proteomic analysis to identify the potential proteins regulated by ROCK2. Notably, Drp1 levels were significantly altered in shROCK2-Pemigatinib-resistant CCA cells (Fig. 4A, B). Drp1 is a key protein that regulates mitochondrial fission and is closely related to ROS production, which may be involved in ferroptosis. Building on these findings, we compared RBE-R and QBC-939-R cells with their parental counterparts (RBE and QBC-939) and found that the resistant cells displayed high Drp1 levels (Fig. 4C). The IHC results indicated that the frequency of Drp1-positive cells was higher in CCA tissues than that in the adjacent non-tumour tissues (Fig. S7A). Additionally, the reduction in Drp1 levels did not lead to significant changes in ROCK2 levels, suggesting that Drp1 may be a downstream target of ROCK2 (Fig. 4D). Furthermore, Drp1-knockdown in RBE-R and QBC-939-R cells enhanced the sensitivity of these cells to Pemigatinib treatment (Figs. 4E–G and S7B–D). Thus, our results indicated that high levels of Drp1 in CCA cells contributed to Pemigatinib resistance in these cells.

Next, we analysed the relationship between ROCK2 and Drp1 expression levels. The western blot analysis results revealed that a reduction in ROCK2 levels lead to a decrease in Drp1 levels in RBE-R and QBC-939-R cells (Fig. 4H). Furthermore, we examined the expression of ROCK2 and Drp1 in 68 tumour-adjacent and 282 CCA tissues, including 58 iCCAs, 123 pCCAs, and 101 dCCAs, using IHC analysis. ROCK2 expression was positively correlated with Drp1 expression in CCA samples (Fig. 4I–L). High expression levels of ROCK2 and Drp1 were associated with poor prognoses in patients with iCCA, pCCA, and dCCA. The patients were further stratified according to their ROCK2 and Drp1 expression patterns. Notably, those showing co-expression of ROCK2 and Drp1 were grouped together; compared to the results obtained based on ROCK2 or Drp1 levels independently, the simultaneous expression of ROCK2 and Drp1 served as an independent risk factor and a more sensitive indicator of poor prognosis across all CCA subtypes (Fig. 4M–P). Our findings indicated that ROCK2 regulates ferroptosis in CCA cells by modulating Drp1 expression, and high levels of both proteins were linked to poor patient prognosis and resistance to Pemigatinib treatment.

### ROCK2 regulates ferroptosis and leads to Drp1-dependent resistance to Pemigatinib in CCA cells

**Fig. 5:**
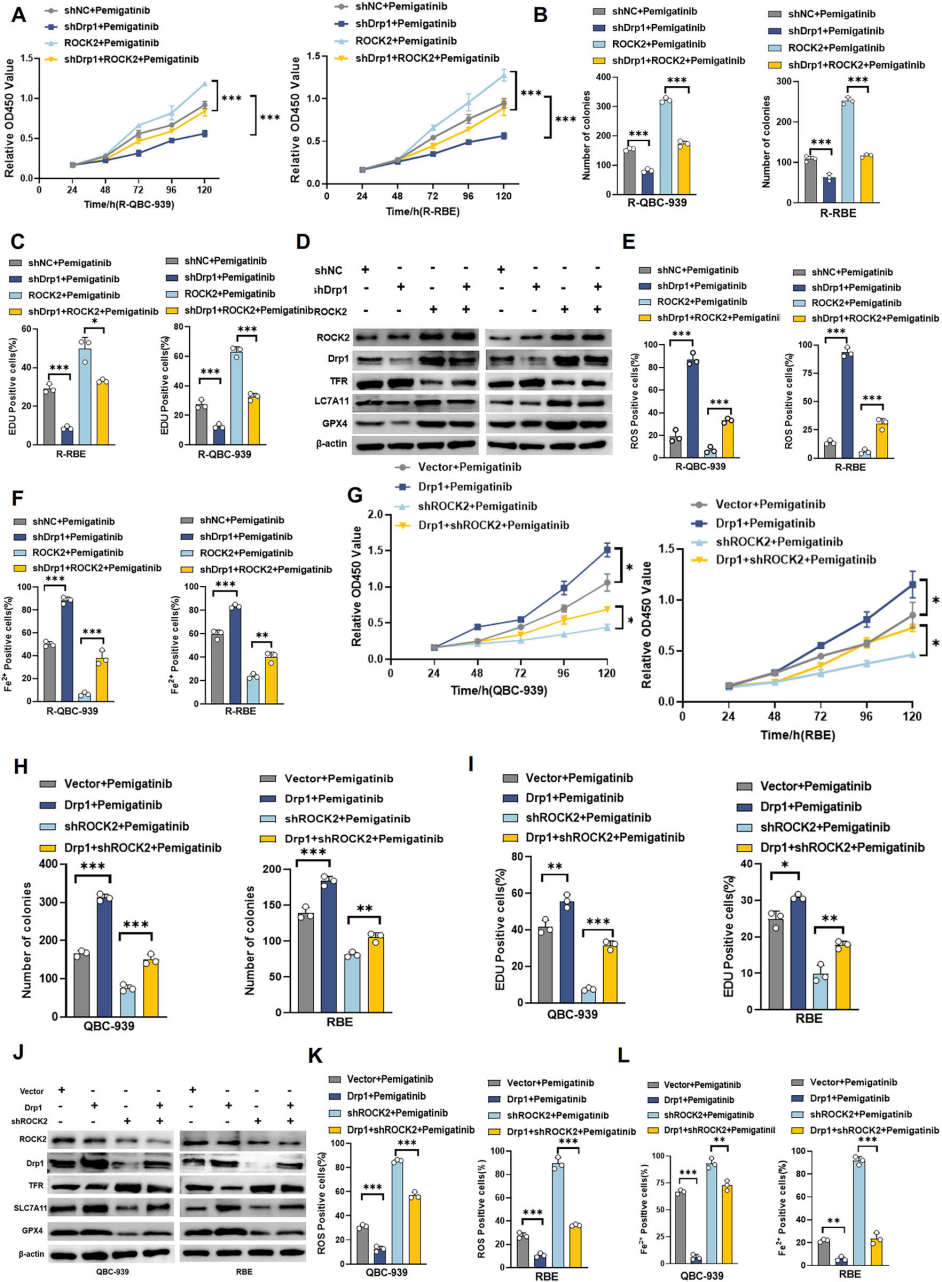
ROCK2 affects ferroptosis and leads to Drp1-dependent resistance to pemigatinib in Cholangiocarcinoma cells. **A**–**C** Cell proliferation was measured using CCK-8 assay, colony formation assay, and EdU assay in R-QBC-939 and R-RBE cells from the indicated groups after treatment with Pemigatinib (5 μM, 48 h at 37 °C). Data indicated as mean ± SD. ***P < 0.001, **P < 0.01. **D** The expression of ROCK2, Drp1, SLC7A11, GPX4, and TFR was detected by western blot in the indicated groups of R-QBC-939 and R-RBE cells. **E**, **F** Intracellular ROS levels and ferrous ion activity were measured in R-QBC-939 and R-RBE cells from the indicated groups after treatment with Pemigatinib (5 μM, 48 h at 37 °C). Data indicated as mean ± SD. ***P < 0.001, **P < 0.01. **G**–**I** Cell proliferation was measured using CCK-8 assay, clone formation assay, and EdU assay in QBC-939 and RBE cells from the indicated groups after treatment with Pemigatinib (5 μM, 48 h at 37 °C). Data indicated as mean ± SD.***P < 0.001, **P < 0.01, *P < 0.05. **J** The expression of ROCK2, Drp1, SLC7A11, GPX4, and TFR was detected by western blot in the indicated groups of QBC-939 and RBE cells. **K**, **L** Intracellular ROS levels and ferrous ion activity were measured in QBC-939 and RBE cells from the indicated groups after treatment with Pemigatinib (5 μM, 48 h at 37 °C). Data indicated as mean ± SD. ***P < 0.001, **P < 0.01.

To determine whether Drp1 is a key protein regulating ROCK2-induced ferroptosis, which leads to the resistance of CCA cells to Pemigatinib, we decreased Drp1 levels in ROCK2-overexpressing cells and observed changes in resistance to Pemigatinib using CCK-8 assays. ROCK2-overexpression increased the proliferation of resistant cells, whereas a decrease in Drp1 levels blocked this process (Fig. 5A). The colony formation assay results showed that the ability of cells to form colonies was significantly diminished in the shDrp1 + ROCK2 group compared to that in the ROCK2 group, reinforcing the notion that Drp1 is critical for maintaining the proliferative ability of ROCK2-overexpressing cells (Figs. 5B and S8A). The EdU incorporation assay was performed to assess cell proliferation based on the amount of DNA synthesised; the EdU-positive cell numbers were significantly reduced when Drp1 was downregulated in ROCK2-overexpressing cells (Figs. 5C and S8B, C). To confirm that Drp1 contributes to the regulation of ferroptosis by ROCK2, we reduced Drp1 expression levels in ROCK2-overexpressing QBC-939-R cells and observed the changes in ferroptosis in drug-resistant cells. The western blot analysis results showed that ROCK2-overexpression decreased the expression levels of TFR and significantly increased the GPX4 and SLC7A11 levels; this regulatory effect of ROCK2 was effectively counteracted by the knockdown of Drp1 (Fig. 5D). Our results indicated that ROCK2-overexpression inhibited ferroptosis and suppression of Drp1 expression blocked this ROCK2-mediated inhibition of ferroptosis (Figs. 5E, F, S8D–F, and S9A–C).

Next, we increased the expression levels of Drp1 in ROCK2-knockdown CCA cells and subsequently assessed the levels of ferroptosis-related proteins and cell proliferation ability using colony formation, CCK-8, EdU incorporation, and western blotting assays. As illustrated in Figs. 5G–I and S8D–F, ROCK2-knockdown decreased CCA cell proliferation, whereas the upregulation of Drp1 expression inhibited the decrease in proliferation induced by ROCK2-knockdown. Immunoblot analysis results showed that ROCK2 knockdown increased TFR levels and significantly decreased GPX4 and SLC7A11 levels; this regulatory effect of ROCK2 was effectively counteracted by Drp1 overexpression (Fig. 5J). Furthermore, ROCK2 knockdown promoted ferroptosis, and Drp1 overexpression blocked this process. Inhibition of ferroptosis significantly rescued cell death induced by ROCK2 knockdown and Drp1 modulation, whereas none of the other inhibitors (Z-VAD, Ne, or 3-MA) showed a comparable effect (Figs. 5K, L, S9G, and S10A–F). These results showed that ROCK2 regulates ferroptosis in CCA cells in a Drp1-dependent manner.

### ROCK2 stabilises the expression of Drp1 by competitively binding with UBA52

**Fig. 6:**
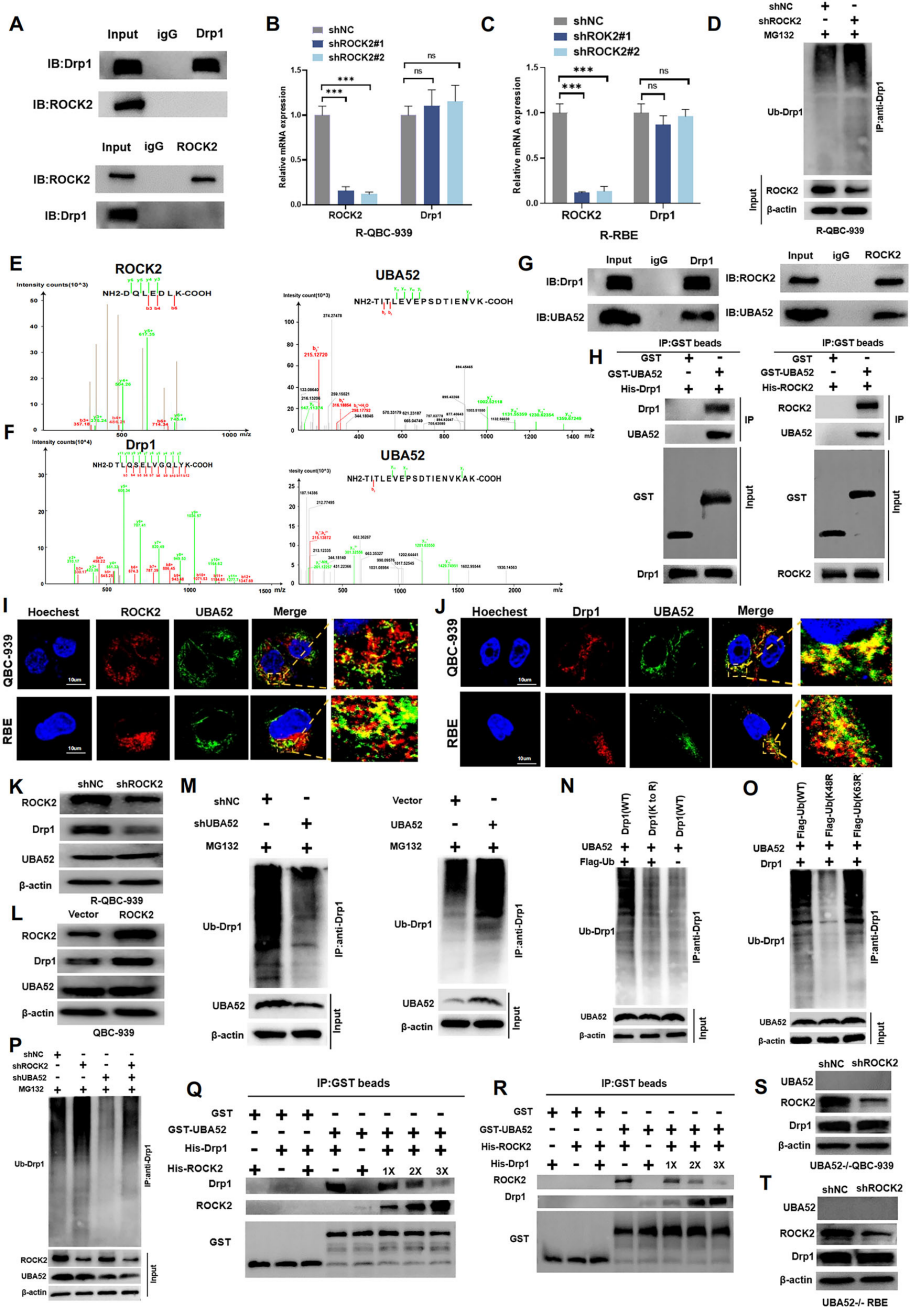
ROCK2 stabilises the expression of Drp1 by competitively binding with UBA52. **A** Co-IP analysis of the interaction between ROCK2 and Drp1 in QBC-939 cells. **B**, **C** qRT-PCR analysis of Drp1 expression was performed in ROCK2 knockdown R-QBC-939 and R-RBE cells. (***P < 0.001, ns, nonsignificant). **D** The ubiquitination of Drp1 was detected by western blot in control and ROCK2 knockdown R-QBC-939 cells treated with MG132 (5 μM, 12 h at 37 °C). **E** The peptide information of UBA52 was obtained from the mass spectrometry (MS) analysis of ROCK2. **F** The peptide information of UBA52 was obtained from the MS analysis of of Drp1. **G** Co-IP analysis of ROCK2-UBA52 and Drp1-UBA52 interactions in QBC-939 cells. **H** GST pull-down assay to detect ROCK2-UBA52 and Drp1-UBA52 interactions in QBC-939 cells. **I** The localization of ROCK2 and UBA52 in the indicated cells were detected by immunofluorescence. Scale bar: 10 μm. **J** The localization of Drp1 and UBA52 in the indicated cells were detected with immunofluorescence. Scale bar: 10 μm. **K**, **L** The expression of ROCK2, Drp1, and UBA52 was detected by western blot in ROCK2-knockdown and ROCK2-overexpressing cells. **M** The ubiquitination of Drp1 was detected by Western blot in UBA52-knockdown R-QBC-939 and UBA52-overexpressing QBC-939 cells treated with MG132 (5 μM, 12 h at 37 °C). **N**, **O** QBC- 939 cells were transfected with the indicated plasmids, and Drp1 ubiquitination was detected. K-to-R, mutations in all Lys positions of Drp1, WT wildtype. **P** The ubiquitination of Drp1 was detected by western blot in the indicated groups of R-QBC-939 cells treated with MG132 (5 μM, 12 h at 37 °C). **Q**, **R** CCA cells were transfected with increasing amounts of the ROCK2 or Drp1 plasmid. GST pull-down and western blot analysis for the indicated proteins. **S**, **T** The expression of ROCK2, Drp1, and UBA52 was detected by western blot after silencing ROCK2 in UBA52-knockout QBC-939 and RBE cells.

ROCK2 has been reported to interact with various substrates to exert its effects, including the inhibition of substrate ubiquitination and degradation [23]. To elucidate the mechanism by which ROCK2 regulates Drp1 expression in CCA cells, we first examined whether ROCK2 and Drp1 directly interacted with each other. The coimmunoprecipitation (co-IP) analysis results revealed no direct interactions between these proteins (Fig. 6A). Moreover, the qRT-PCR results showed that ROCK2 expression did not affect the mRNA levels of Drp1 (Figs. 6B, C). However, the ectopic expression of ROCK2 lead to a substantial decrease in Drp1 polyubiquitination, whereas ROCK2 knockdown increased Drp1 polyubiquitination (Figs. 6D and S11A). Furthermore, our results showed that after MG132 treatment of CCA cells, the decrease of ROCK2 level had no significant effect on Drp1 expression, while after lysosome inhibitor Leupetin treatment of CCA cells, the decrease of ROCK2 level would still lead to the decrease of Drp1 expression (Fig. S10B, C). Moreover, the degradation assay results revealed that the half-life of exogenously expressed Drp1 was significantly decreased in ROCK2-overexpressing CCA cells compared to that in control cells (Fig. S11D, E). Collectively, these data demonstrated that ROCK2 promotes Drp1 ubiquitination and degradation. However, the mechanism by which ROCK2 affects this process remains unclear. Mass spectrometric analysis revealed that ubiquitin A-52 residue ribosomal protein fusion product 1 (UBA52) could bind to both ROCK2 and Drp1, suggesting that UBA52 is a potential E3 ubiquitin ligase for Drp1 (Fig. 6E, F). Co-IP analysis revealed an interaction between ROCK2 and UBA52 as well as between Drp1 and UBA52 (Figs. 6G and S11F, G). The purified GST–UBA52 complex bound to His-tagged ROCK2 and Drp1 in vitro (Fig. 6H). Findings of confocal microscopic analysis showed that UBA52 was co-localised with ROCK2 and Drp1 in the cytoplasm (Fig. 6I, J). These results suggested that ROCK2 regulated Drp1 expression via UBA52.

No significant changes in UBA52 protein levels were observed after altering ROCK2 expression levels (Fig. 6K, L). UBA52 downregulated Drp1 protein levels (Fig. S10H, I). However, UBA52-overexpression enhanced the ubiquitination level of Drp1; a decrease in UBA52 levels inhibited the ubiquitination of Drp1 (Fig. 6M). Furthermore, the data showed that mutations at all Lys residues of Drp1 inhibited Drp1 polyubiquitination (Fig. 6N). Additionally, mutation at the Lys48 position of ubiquitin substantially inhibited UBA52-mediated Drp1 ubiquitination, whereas the K63R mutation in ubiquitin had no effect on Drp1 ubiquitination (Fig. 6O). These results indicated that UBA52 promoted Drp1 degradation via the ubiquitin–proteasome pathway in CCA cells. Additionally, a decrease in ROCK2 levels was observed to increase Drp1 ubiquitination, and a decrease in UBA52 levels blocked this process (Fig. 6P). ROCK2-overexpression inhibited Drp1 ubiquitination, and UBA52-overexpression blocked this process (Fig. S10J). Therefore, we speculated that ROCK2 may inhibit the binding of UBA52 to Drp1 by interacting with UBA52. The results of in vitro protein competition experiments revealed that a gradual increase in ROCK2 levels enhanced ROCK2–UBA52 complex formation and decreased UBA52–Drp1 complex formation. Conversely, an increase in Drp1 levels in the cells lead to a reduction in ROCK2–UBA52 complex levels and an increase in Drp1–UBA52 complex levels (Fig. 6Q, R). The Drp1 levels remained unaffected following ROCK2-silencing in UBA52-knockout cells (Fig. 6S, T). The findings suggested that ROCK2 and Drp1 competitively bind to UBA52; ROCK2-overexpression promotes ROCK2–UBA52 complex formation and inhibits Drp1–UBA52 complex formation, which enhanced Drp1 expression levels.

### ROCK2-mediated inhibition of ferroptosis leads to UBA52–Drp1 axis-dependent Pemigatinib resistance in Cholangiocarcinoma cells

**Fig. 7:**
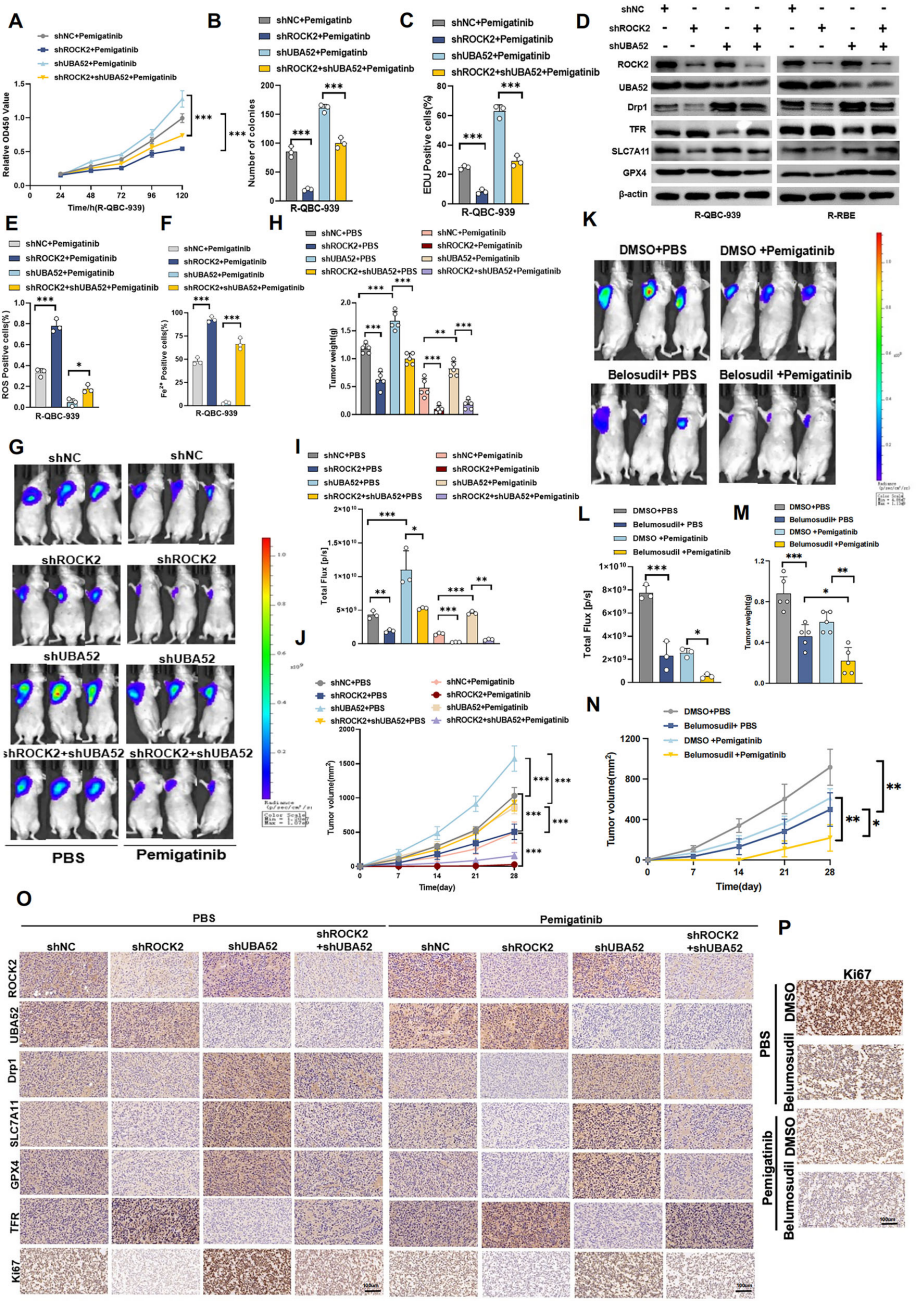
ROCK2-mediated inhibition of ferroptosis leads to UBA52–Drp1 axis-dependent pemigatinib resistance in Cholangiocarcinoma cells. **A**–**C** Cell proliferation was measured using CCK-8 assay, clone formation assay, and EdU assay in R-QBC-939 cells from the indicated groups after treatment with Pemigatinib (5 μM, 48 h at 37 °C). Data indicated as mean ± SD. ***P < 0.001. **D** The expression of ROCK2, Drp1, UBA52, SLC7A11, GPX4, and TFR was detected by western blot in the indicated groups of R-QBC-939 and R-RBE cells. **E**, **F** Intracellular ROS levels and ferrous ion activity were measured in R-QBC-939 cells from the indicated groups after treatment with Pemigatinib (5 μM, 48 h at 37 °C). Data indicated as mean ± SD. ***P < 0.001, **P < 0.01,*P < 0.05. **G**, **H** In vivo tumor formation was examined by subcutaneously injecting QBC-939 cells treated with the indicated protocols into the flanks of nude mice administered Pemigatinib (10 mg/kg). Bioluminescence imaging was performed 28 days post inoculation (*n* = 3). ***P < 0.001, **P < 0.01. **I**, **J** Tumour weight was measured at the end of the experiment, while tumour volume was monitored every 7 days, allowing the corresponding tumour growth curves to be plotted. Data indicated as mean ± SD (*n* = 5). ***P < 0.001, **P < 0.01, *P < 0.05. **K**, **L** In vivo tumor formation was examined by subcutaneously injecting R-QBC-939 cells treated with Pemigatinib(10 mg/kg) and Belosudil(50 mg/kg) into the flanks of nude mice. Bioluminescence imaging was performed 28 days post-inoculation (*n* = 3). Data indicated as mean ± SD. ***P < 0.001, *P < 0.05. **M**, **N** Tumour weight was measured at the end of the experiment, while tumour volume was monitored every 7 days, allowing the corresponding tumour growth curves to be plotted (*n* = 5). Data indicated as mean ± SD.***P < 0.001, **P < 0.01. **O** Representative IHC staining of ROCK2, UBA52, Drp1,GPX4, TFR, SLC7A11 and Ki67 in allograft tumor tissues (*n* = 5). Scale bar: 100 μm. **P** Representative IHC staining of Ki67 in allograft tumor tissues (*n* = 5). Scale bar: 100 μm.

Finally, we performed rescue experiments to determine whether ROCK2-mediated inhibition of ferroptosis leads to Pemigatinib resistance in CCA cells, which is dependent on the UBA52–Drp1 axis. ROCK2 knockdown significantly decreased CCA cell proliferation, and the effect was mitigated by the concurrent knockdown of UBA52 (Figs. 7A, B and S12A–C). EdU assay results revealed that a decrease in ROCK2 levels reduced the proliferation of drug-resistant CCA cells treated with Pemigatinib, whereas UBA52-knockdown restored the proliferative capacity of these drug-resistant cells (Fig. 7C and S12D–F). Western blot analysis revealed that ROCK2 knockdown increased TFR levels and significantly decreased Drp1, GPX4 and SLC7A11 levels. The regulatory effect of ROCK2-knockdown was effectively counteracted by the knockdown of UBA52 (Fig. 7D). The results indicated that ROCK2-knockdown promoted ferroptosis, and suppression of UBA52 expression blocked this ROCK2-mediated enhancement of ferroptosis (Figs. 7E, F, S12G–H, and S13A–G). The reverse effect was also observed ROCK2-overexpression suppression ferroptosis, and overexpression of UBA52 expression blocked this ROCK2-mediated weaken of ferroptosis (Figs. S13H–K, S14A–G, and S15A–F).

**Fig. 8:**
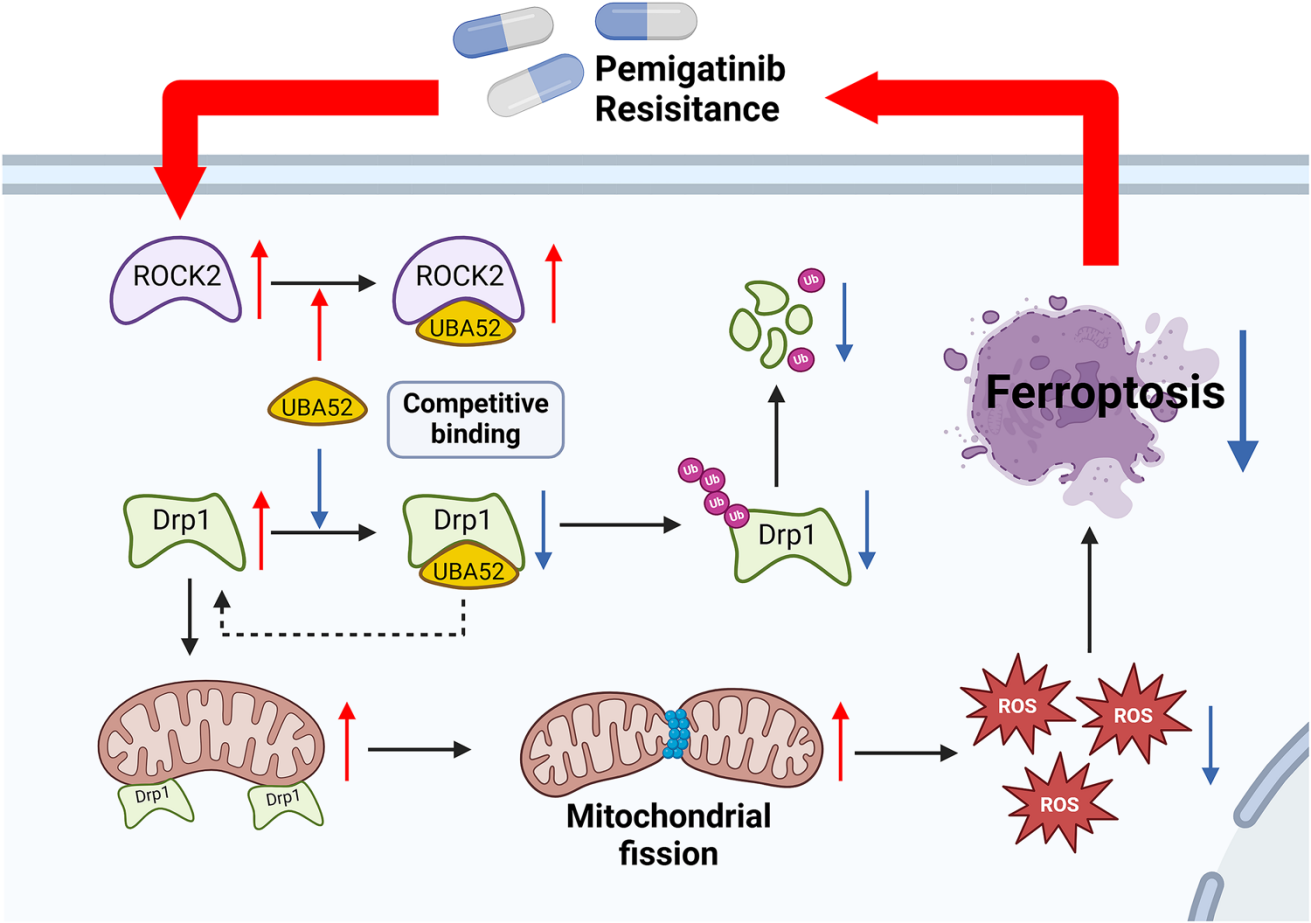
A cartoon summarizing our findings. The proposed mechanistic scheme suggests that ROCK2 competitively binds to UBA52, the E3 ubiquitin ligase of Drp1, stabilizing Drp1 expression and thereby inhibiting ferroptosis, which leads to pemetinib resistance in cholangioma.

We subcutaneously implanted CCA cells, in which either ROCK2 alone was knocked down or ROCK2 and UBA52 were knocked down, into nude mice to monitor tumour growth. As shown in Figs. 7G–J and S15G, H, mice harbouring ROCK2-silenced cells exhibited a notable decrease in tumour weight and volume; however, the simultaneous knockdown of ROCK2 and UBA52 completely abolished the antitumour effect of Pemigatinib in ROCK2-knockdown cells; no significant difference in the total weights of the mice was observed before and after cell implantation. The ROCK2 inhibitor—belumosudil—significantly enhanced the sensitivity of CCA cells to pemigatinib in vivo (Fig. 7K–N and S15I, J). By modulating ROCK2 and UBA52, and using ferroptosis inhibitor FDO, apoptosis inhibitor Z-VAD, necrosis inhibitor Ne, and autophagy inhibitor 3-MA, we demonstrated that ROCK2-UBA52 influences pemetrexed resistance through ferroptosis, with no involvement of other cell death pathways(Fig. S15K). In line with these data, the IHC assay results showed an increase in TFR intensity and a decrease in Drp1 levels and Ki67-positive proliferative cells in ROCK2-knockdown tumours. Concomitant knockdown of UBA52 reversed these effects (Fig. 7O). Moreover, the IHC assay results revealed that treatment of cells with a combination of belumosudil and Pemigatinib significantly reduced the number of Ki67-positive proliferative cells (Fig. 7P). These results suggested that ROCK2-mediated inhibition of ferroptosis contributes to Pemigatinib resistance in CCA cells through the UBA52–Drp1 axis; targeting of ROCK2 with the inhibitor—belumosudil—enhanced the sensitivity of these cancer cells to Pemigatinib (Fig. 8).

## Discussion

CCA is characterised by insidious onset, high malignancy, rapid progression, and poor prognosis and is largely insensitive to adjuvant therapies such as chemotherapy and radiotherapy [24]. In 2020, Pemigatinib became the first targeted therapy approved for the treatment of CCA [25]. However, cases of Pemigatinib resistance have been reported, indicating that not all patients benefit from this treatment [5–7]. Therefore, it is crucial to understand the mechanisms underlying Pemigatinib resistance as this knowledge can guide clinical decision-making and lead to the identification of new therapeutic targets. Ultimately, addressing Pemigatinib resistance could improve the sensitivity of CCA cells to treatment, thereby enhancing patient outcomes and quality of life. The findings presented in this study elucidate the critical role of ROCK2 in the development of Pemigatinib resistance in CCA through the modulation of ferroptosis, particularly highlighting the involvement of the UBA52–Drp1 axis. Our results demonstrated that ROCK2 is not only a biomarker for poor prognosis in CCA but also a potential therapeutic target that can be exploited to enhance the efficacy of Pemigatinib.

ROCK2 is a crucial signalling molecule in the Rho/ROCK signalling pathway [26, 27]. Recent studies have established a connection among ROCK2, tumour development, and drug resistance [18, 28–30]. However, the role that ROCK2 plays in Pemigatinib resistance in CCA cells remains unexplored. To the best of our knowledge, this is the first study to investigate ROCK2 expression in this context. We observed that ROCK2 was overexpressed in CCA cells, and high ROCK2 levels were associated with poor prognosis in patients with CCA. Notably, ROCK2 was highly expressed in Pemigatinib-resistant CCA cell lines, and its expression was positively correlated with Pemigatinib resistance. The results of in vivo and in vitro experiments revealed that a decrease in ROCK2 expression levels promoted ferroptosis in CCA cells, making them more sensitive to Pemigatinib treatment. Additionally, we found that the ROCK2 inhibitor—belumosudil—significantly increased the sensitivity of CCA cells to Pemigatinib. Moreover, based on our results, we demonstrate that ROCK2 plays a critical role in regulating pemetrexed resistance in CCA cells through the ferroptosis pathway, with no involvement of other forms of cell death. Importantly, inhibition of ferroptosis using the ferroptosis inhibitor FDO significantly reduced the cell death induced by ROCK2 knockdown, while inhibitors of apoptosis (Z-VAD), necrosis (Ne), and autophagy (3-MA) had no comparable effect, suggesting that the mechanism is ferroptosis-specific. This finding corroborates those of previous studies, indicating that ROCK2 plays a critical role in inhibiting ferroptosis, which in turn affects the resistance of CCA cells to Pemigatinib.

Drp1 is a crucial protein that regulates mitochondrial fission [31]. Additionally, Drp1 is closely associated with the production of ROS, which are the byproducts of cellular metabolism and play significant roles in various cellular signalling pathways [32, 33]. In the context of ferroptosis, a form of regulated cell death characterised by the accumulation of lipid peroxides [34, 35], the role of Drp1 becomes particularly important. ROS production can lead to oxidative stress, which is a key trigger for ferroptosis [36, 37]. Drp1-mediated mitochondrial fission enhances ROS production, thereby promoting conditions that favour ferroptosis [38]. Drp1 can induce ferroptosis in various tumours [39, 40]. For example, Drp1-downregulation significantly inhibits the metastatic ability and ferroptosis of breast cancer cells [41]. Drp1 induces ferroptosis and enhances the invasion of HCC cells [42]. Moreover, Drp1 has been implicated in chemotherapy resistance. For instance, CRL4 can recruit Drp1 to mitochondria to induce mitophagy and inhibit chemotherapy resistance of ovarian cancer [43]. PARP14 induces Drp1 expression to increase mitophagy and drug resistance in multiple myeloma [44]. However, whether Drp1 regulates ferroptosis-mediated resistance to targeted therapies for CCA remains unclear. Our experimental data revealed that the inhibition of Drp1 expression reversed ferroptosis in Pemigatinib-resistant CCA cell lines; however, this reversal was observed in the presence of a ferroptosis activator. Importantly, ROCK2 affected ferroptosis and lead to Drp1-dependent Pemigatinib resistance in CCA cells. In vitro experiments revealed that Drp1-upregulation hindered the changes in ferroptosis-related protein levels caused by ROCK2-knockdown, and Drp1-downregulation inhibited ROCK2-overexpression-induced ferroptosis. These results indicated that Drp1 is a key factor in the ROCK2-mediated inhibition of ferroptosis in CCA cells. Therefore, targeting of the ROCK2–Drp1 axis could be a potential therapeutic strategy to overcome chemotherapeutic resistance in CCA cells.

Finally, we investigated the mechanism by which ROCK2 regulates Drp1 expression in CCA cells. ROCK2 interacts with various substrates to exert its effects, including the inhibition of substrate ubiquitination and degradation. Our previous research showed that ROCK2 promoted the proliferation of HCC cells by inhibiting the ubiquitination and degradation of CDC25A [45]. Similarly, ROCK2 enhanced the invasion and metastasis of HCC cells by modulating the ubiquitination and degradation of matrix metalloproteinase 2 (MMP2) [46]. Additionally, ROCK2 promoted osteosarcoma growth and metastasis by modulating the ubiquitination and degradation of PFKFB3 [23]. Collectively, these findings confirmed that ROCK2 can stabilise substrate proteins by inhibiting their ubiquitination and degradation. However, the precise mechanism by which ROCK2 inhibits substrate ubiquitination and degradation remains to be elucidated. In our study, ROCK2 increased the expression level of Drp1 by affecting its ubiquitination levels, although no direct interaction between ROCK2 and Drp1 was observed. Through a series of proteomic experiments, we discovered that ROCK2 inhibited the ubiquitination and degradation of Drp1 by competitively binding to UBA52, a novel E3 ubiquitin ligase for Drp1. ROCK2 and Drp1 competitively bound to UBA52. ROCK2-overexpression promoted ROCK2–UBA52 complex formation and decreased Drp1–UBA52 complex formation, ultimately enhancing Drp1 expression levels. This conclusion is supported by several observations. First, co-IP analysis revealed the interaction between ROCK2 and UBA52, as well as that between Drp1 and UBA52. Second, UBA52 is a potential E3 ubiquitin ligase of Drp1. Overexpression of UBA52 enhanced Drp1 ubiquitination, whereas UBA52-downregulation inhibited Drp1 ubiquitination. Third, ROCK2 and Drp1 competitively bound to UBA52, and ROCK2-overexpression increased ROCK2–UBA52 complex formation and decreased Drp1–UBA52 complex formation. Finally, ROCK2-mediated inhibition of ferroptosis lead to Pemigatinib resistance in CCA cells, which was dependent on the UBA52–Drp1 axis. Additionally, by modulating both ROCK2 and UBA52 expression and treating with the ferroptosis inhibitor FDO, we observed that ROCK2-UBA52 interaction directly influences pemetrexed resistance via ferroptosis, with no contribution from apoptosis, necrosis, or autophagy. These findings support the notion that ROCK2 primarily mediates pemetrexed resistance through ferroptosis and is not involved in other forms of cell death, highlighting a novel regulatory pathway that could be targeted for therapeutic strategies in CCA treatment.

In summary, our study highlights the critical role of ROCK2 in mediating Pemigatinib resistance in CCA cells, which is caused by the modulation of ferroptosis via the UBA52–Drp1 axis. This finding emphasises the potential therapeutic implications of targeting the ROCK2–Drp1 pathway to overcome resistance in CCA cells (Fig. 8). By establishing ROCK2 as a central player in the resistance mechanism, our study paves the way for new therapeutic interventions. Further exploration of ROCK2 inhibitors and their combination with Pemigatinib or other ferroptosis-inducing agents could enhance treatment efficacy. Additionally, investigating the broader implications of the ROCK2–Drp1 axis in other cancers may yield insights into the universal mechanisms of resistance. Ultimately, these findings will not only contribute to a better understanding of CCA biology, but will also support the development of effective, personalised treatment strategies for such patients.

## Supplementary information

supplementary materials

## Data Availability

All data in our study are available from the corresponding author upon reasonable request.
