# Supplementary material for: Targeting the ROCK2/UBA52/DRP1 axis enhances ferroptosis and overcomes pemigatinib resistance in Cholangiocarcinoma
Source: Cell Death Dis. 2025 Jul 4;16(1):493. doi: 10.1038/s41419-025-07804-9 (PMC12227600; doi:10.1038/s41419-025-07804-9)
Supplement: Supplementary file 1 — supplementary materials [file 41419_2025_7804_MOESM1_ESM.pdf]

Supplementary Materials

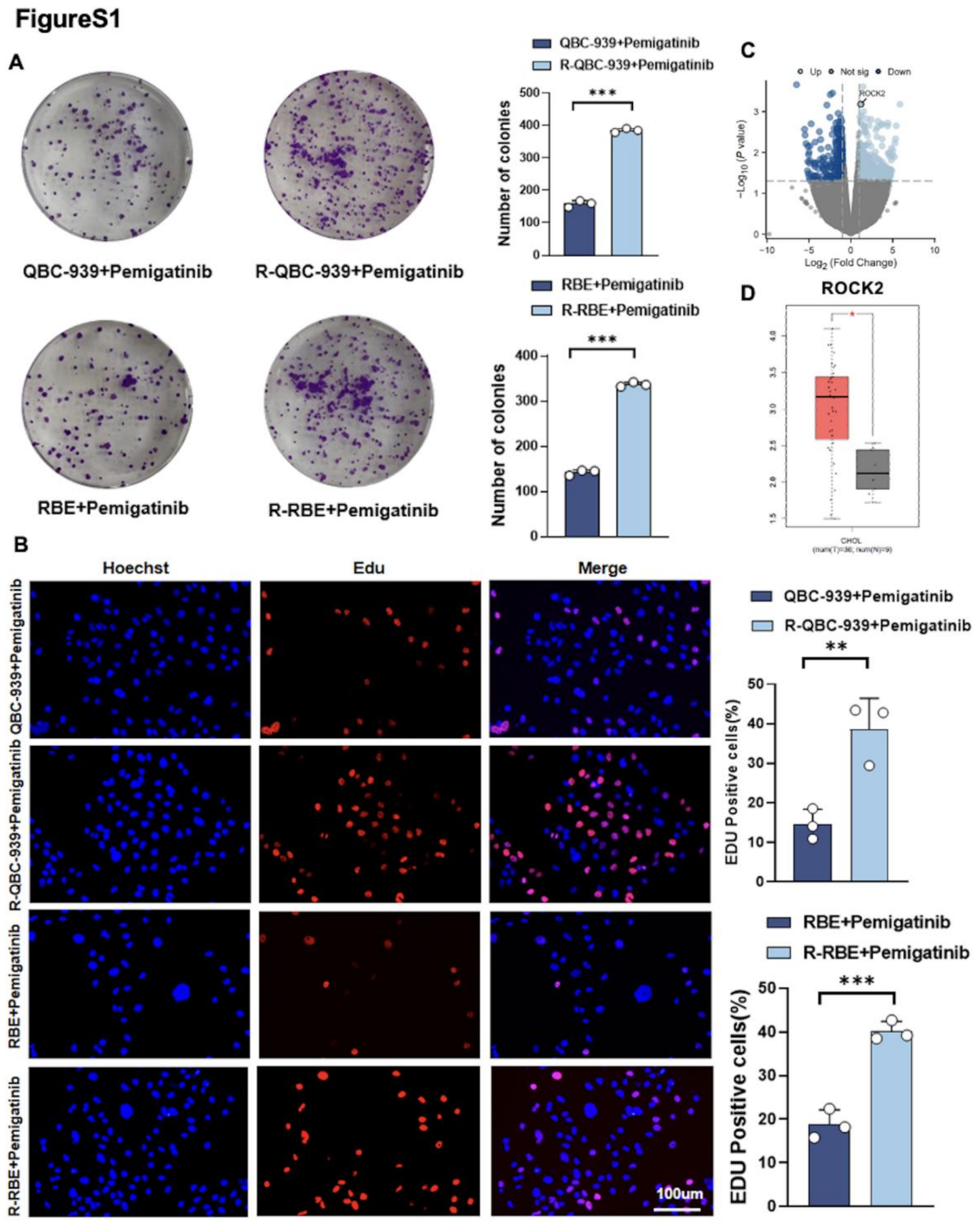

Fig.S1 ROCK2 is strongly expressed in pemigatinib-resistant Cholangiocarcinoma cells and leads to poor prognosis

A.RNA-seq analysis showed differentially expressed genes in pemigatinib-resistant and parental cells.

B. ROCK2 expression in patients with CHOL from the Gene Expression Profiling Interactive Analysis database *P < 0.05.

C-D. Cell proliferation was measured using clone formation assays and EdU assay in QBC-939,RBE and R-QBC-939,R-RBE cells after treatment with Pemigatinib(5 μM, 48h at 37° C) .Data indicated as mean ± S.D.***P < 0.001,**P < 0.01.

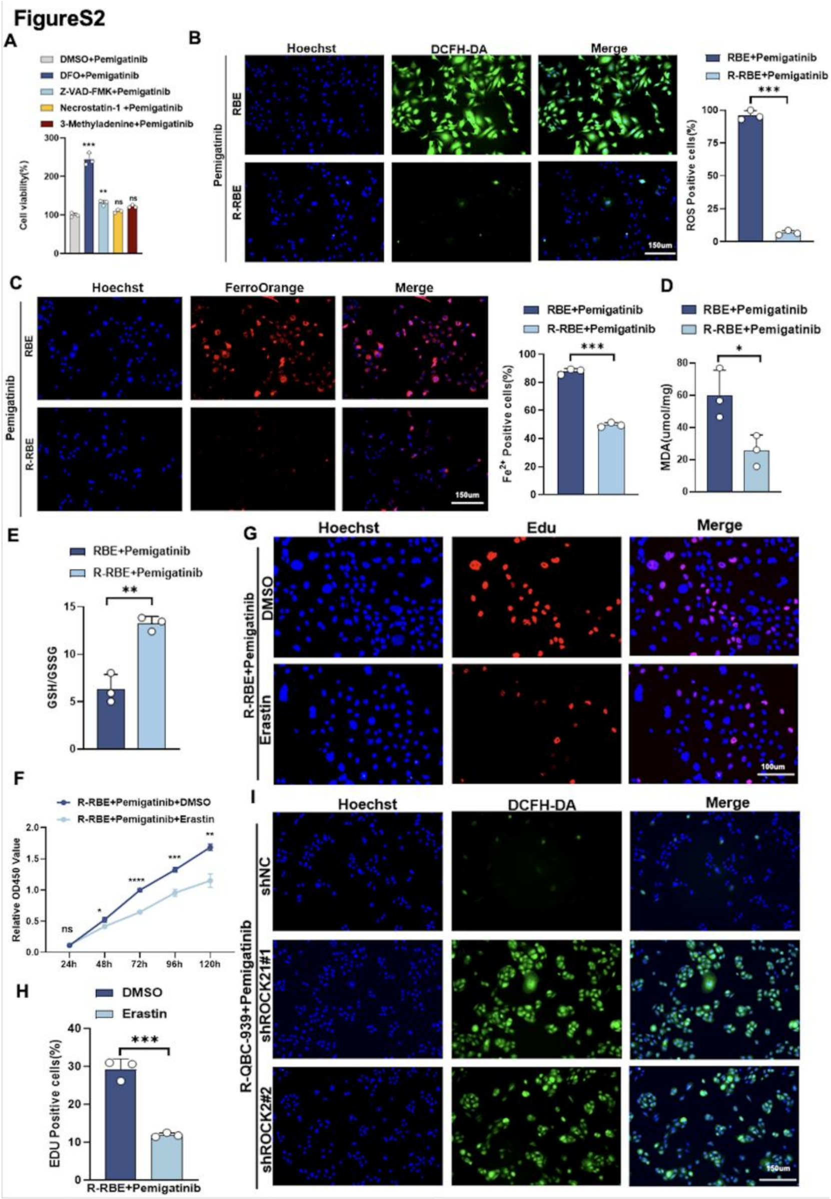

Fig.S2 ROCK2 inhibits ferroptosis and leads to pemigatinib resistance in Cholangiocarcinoma cells

A. Cell proliferation was measured using CCK-8 assay in R-QBC-939 cells with the indicated treatments .Data indicated as mean ± S.D.***P < 0.001,**P < 0.05 , ns, nonsignificant.

B-E.Intracellular ROS levels, ferrous ion activity, MDA content, and GSH/GSSG ratio were measured in RBE and R-RBE cells after treatment with Pemigatinib (5 µM, 48 h at 37 °C).Data indicated as mean ± S.D.***P < 0.001,**P < 0.01,*P < 0.05.

F. Cell proliferation was measured using CCK-8 assay in R-RBE after treatment with Pemigatinib(5uM,48h at 37°C) and and Erastin(2µM,48h at 37°C) .***P < 0.001, **P < 0.01, *P < 0.05, ns,nonsignificant.

G-H. Cell proliferation was measured using EDU assays in R-RBE cells after treatment with Pemigatinib(5uM,48h at 37°C) and and Erastin(2µM,48h at 37°C).Data indicated as mean ± S.D.***P < 0.001.

I. Intracellular ROS levels were measured in control and ROCK2 knockdown R-QBC-939 cellsafter treatment with Pemigatinib (5µM, 48 h at 37°C).

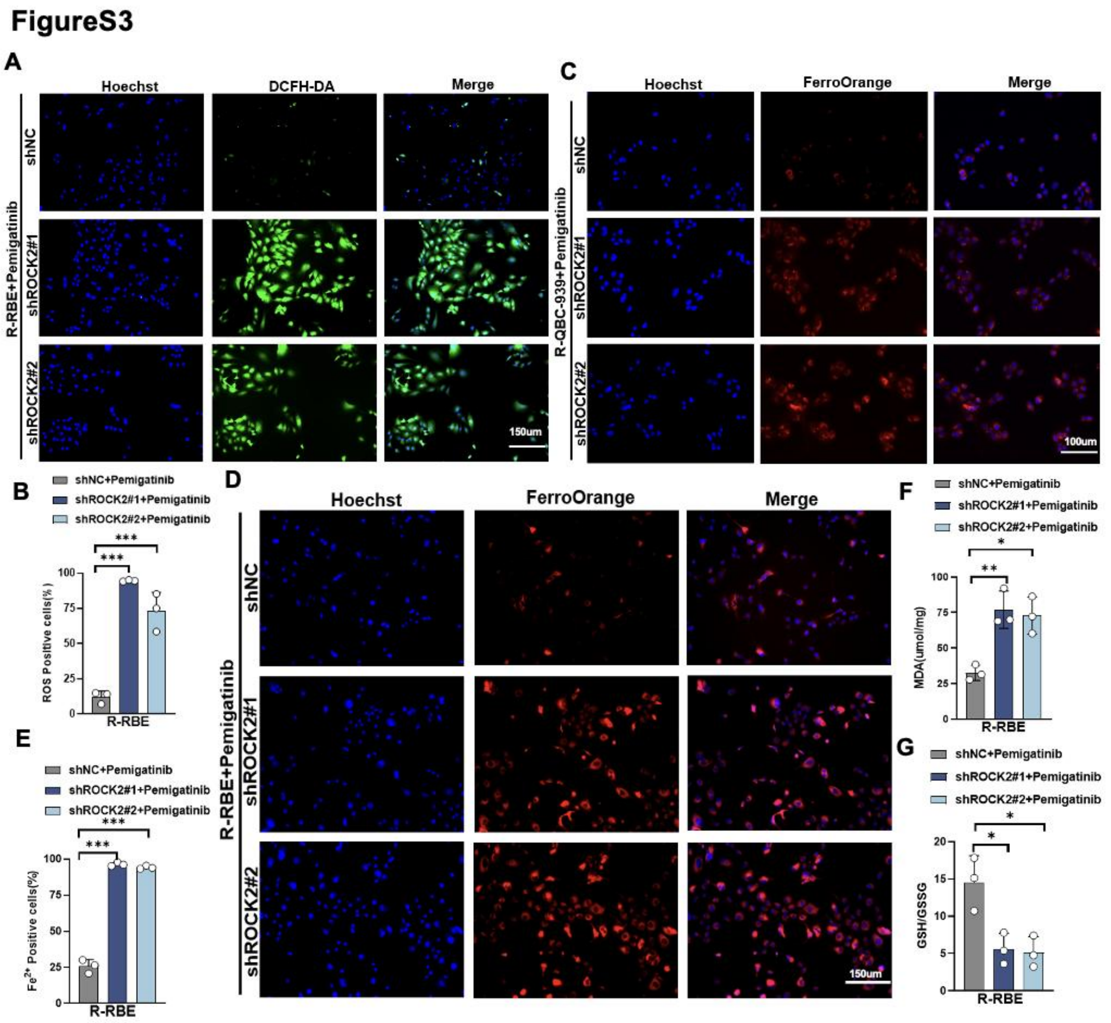

Fig.S3 Ferroptosis was significantly elevated in shROCK2 pemigatinib-resistant CCA cells following pemigatinib treatment

A-G.Intracellular ROS levels, ferrous ion activity, MDA content, and GSH/GSSG ratio were measured in control and ROCK2 knockdown R-RBE cells after treatment with Pemigatinib (5 μM, 48 h at 37°C).Data indicated as mean ± S.D.***$P < 0.001$,**$P < 0.01$,*$P < 0.05$.

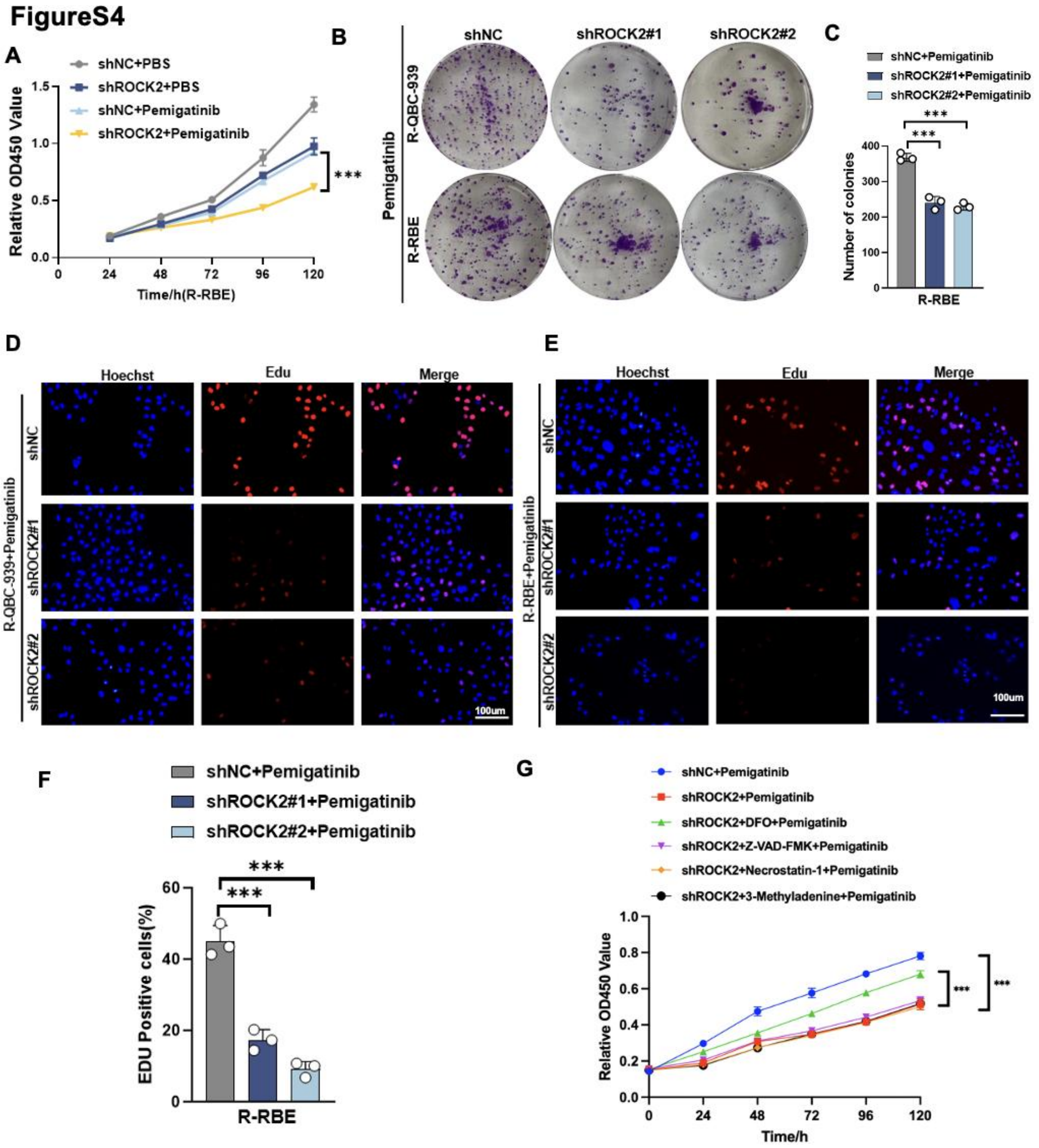

Fig.S4 ROCK2-knockdown in RBE-R and QBC-939-R cells significantly enhanced the sensitivity of these cells to pemigatinib

A. Cell viability was detected using the CCK-8 assay in konckdown ROCK2 R-RBE cells after treatment with Pemigatinib (5 μM, 48 h at 37°C).Data indicated as mean ± S.D.***P < 0.001.

B-C. Cell proliferation was measured using clone formation assays in control and konckdown ROCK2 R-QBC-939 and R-RBE cellsafter treatment with Pemigatinib (5 μM, 48 h at 37°C).Data indicated as mean ± S.D.***P < 0.001.

D-F. Cell proliferation was measured using EDU assays in control and konckdown ROCK2 R-QBC-939 and R-RBE cells after treatment with Pemigatinib (5 μM, 48 h at 37°C).Data indicated as mean ± S.D.***P < 0.001.

G. Cell viability was detected using the CCK-8 assay in indicated groups in R-RBE cells.Data indicated as mean ± S.D.***P < 0.001, **P < 0.01.

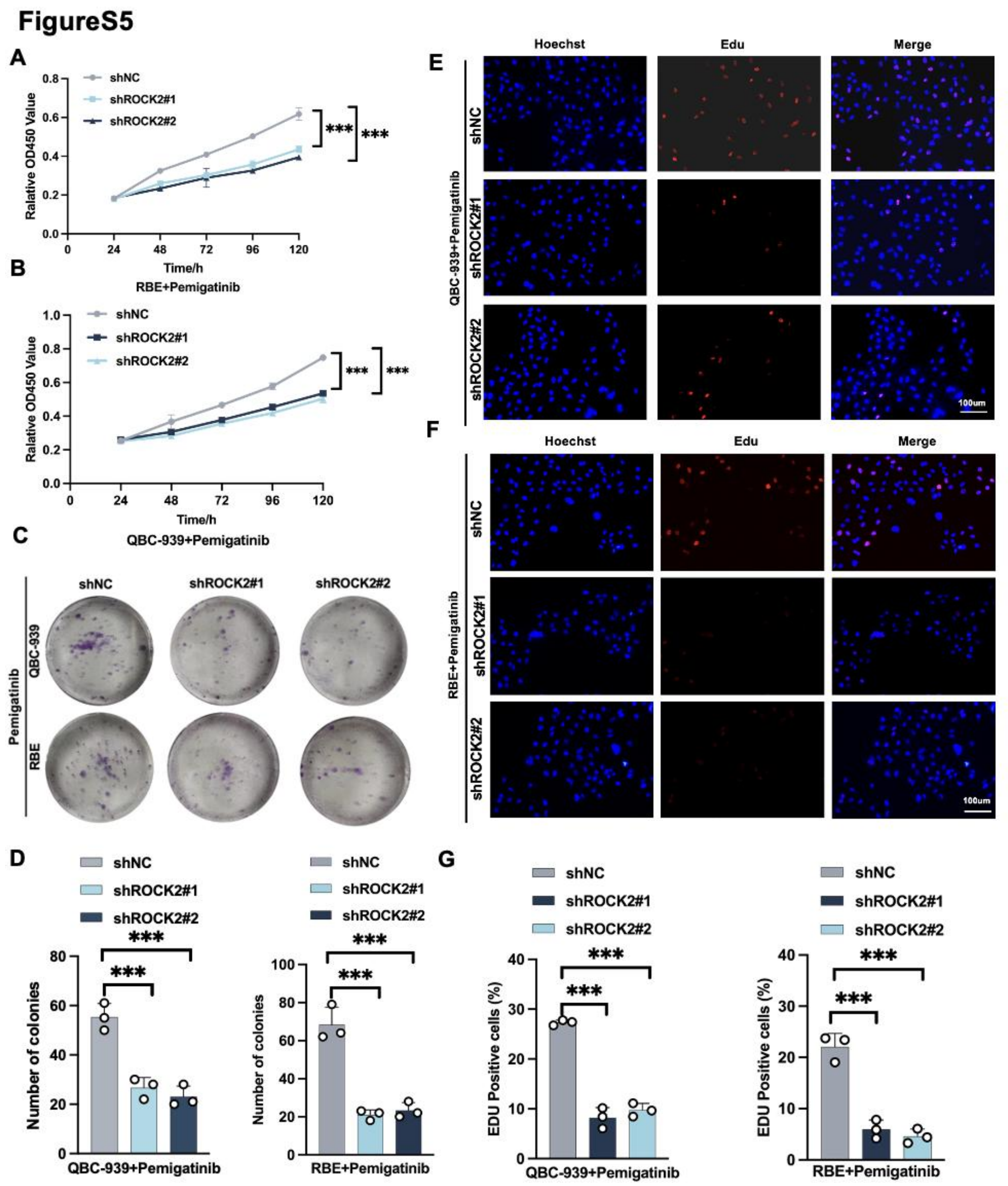

Fig.S5 ROCK2-knockdown in RBE and QBC-939 cells significantly enhanced the sensitivity of these cells to pemigatinib

A-G.Cell proliferation was measured using the CCK-8 assay, clone formation assay, and EdU assay in control and ROCK2-knockdown QBC-939 and RBE cells after treatment with Pemigatinib(5 μM, 48h at 37°C).Data indicated as mean ± S.D.***P < 0.001.

FigureS6

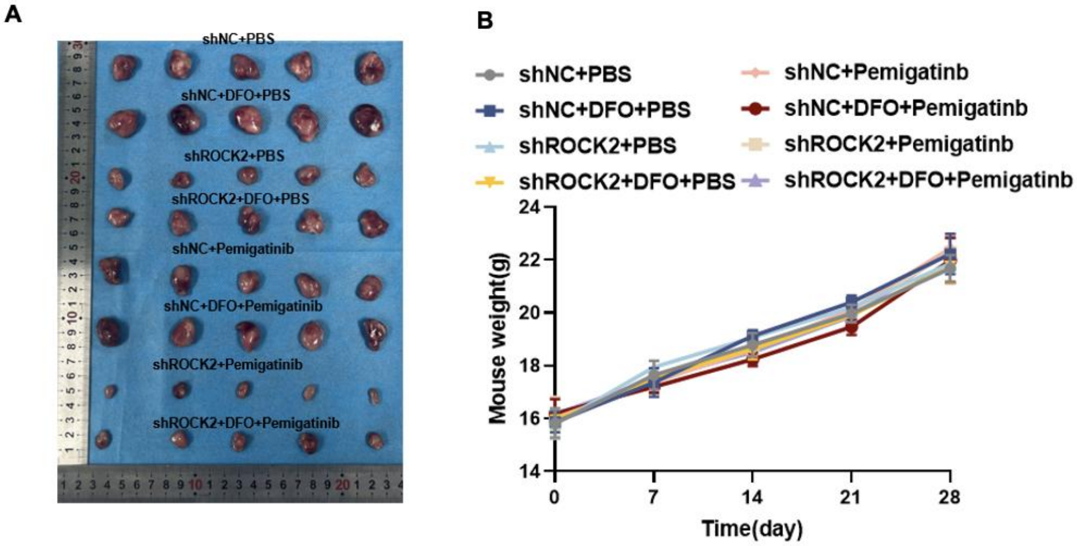

Fig.S6 ROCK2-knockdown increased the sensitivity of CCA cells to pemigatinib, while the addition of DFO blocked this process *in* vivo

A-B. *In vivo* tumour formation was examined by subcutaneous injection of R-QBC-939 shNC or shROCK2 cells subjected to the indicated treatments into the flanks of nude mice and treatment with/without Pemigatinib(10mg/kg) and Deferoxamine(20mg/kg). Representative photographs were obtained 28 days after inoculation. Mice weight was measured and the corresponding Mice weight growth curves were obtained(n=5).

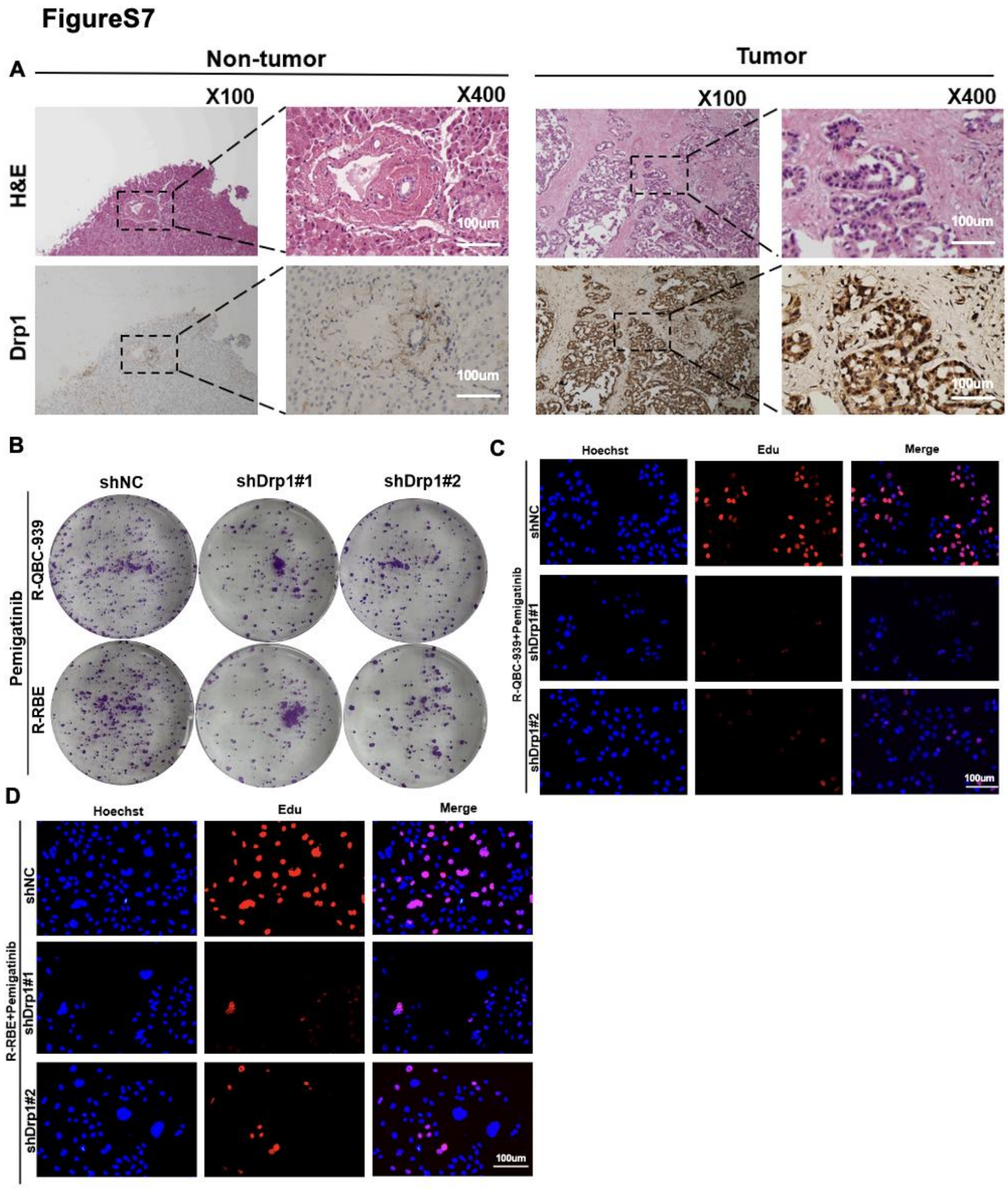

Fig.S7 ROCK2 regulates Drp1 expression and ROCK2 and Drp1 expression levels are positively correlated in CCA tissues

A.Representative immunohistochemistry staining of Drp1 in CCA tissues.

B.Cell proliferation was measured using clone formation assays in control and konckdown Drp1 R-QBC-939 and R-RBE cells after treatment with Pemigatinib(5 μM, 48h at 37°C).

C-D. Cell proliferation was measured using EDU assays in control and konckdown Drp1 R-QBC-939 and R-RBE cells after treatment with Pemigatinib(5 μM, 48h at 37°C).

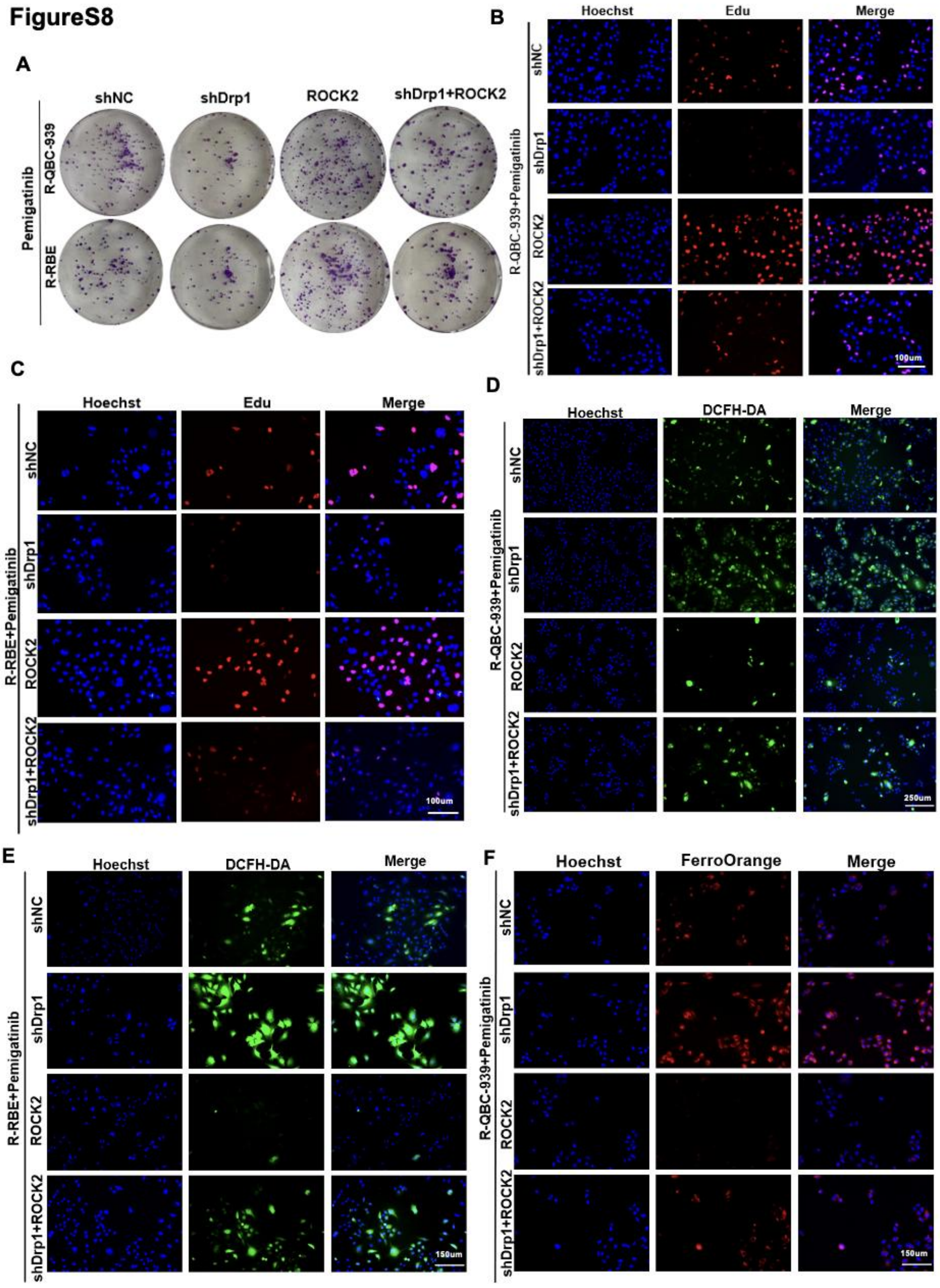

Fig.S8 ROCK2 regulates ferroptosis and leads to Drp1-dependent resistance to pemigatinib in CCA cells

A.Cell proliferation was measured using clone formation assays in indicated groups in R-QBC-939 and R-RBE cells.

B-C. Cell proliferation was measured using EDU assays in indicated groups in R-QBC-939 and R-RBE cells.

D-E. Intracellular ROS levels were measured in R-QBC-939 and R-RBE cells after treatment with Pemigatinib(5 μM, 48h at 37°C).

F. Intracellular ferrous ion activity was measured in R-QBC-939 cells after treatment with Pemigatinib(5 μM, 48h at 37°C).

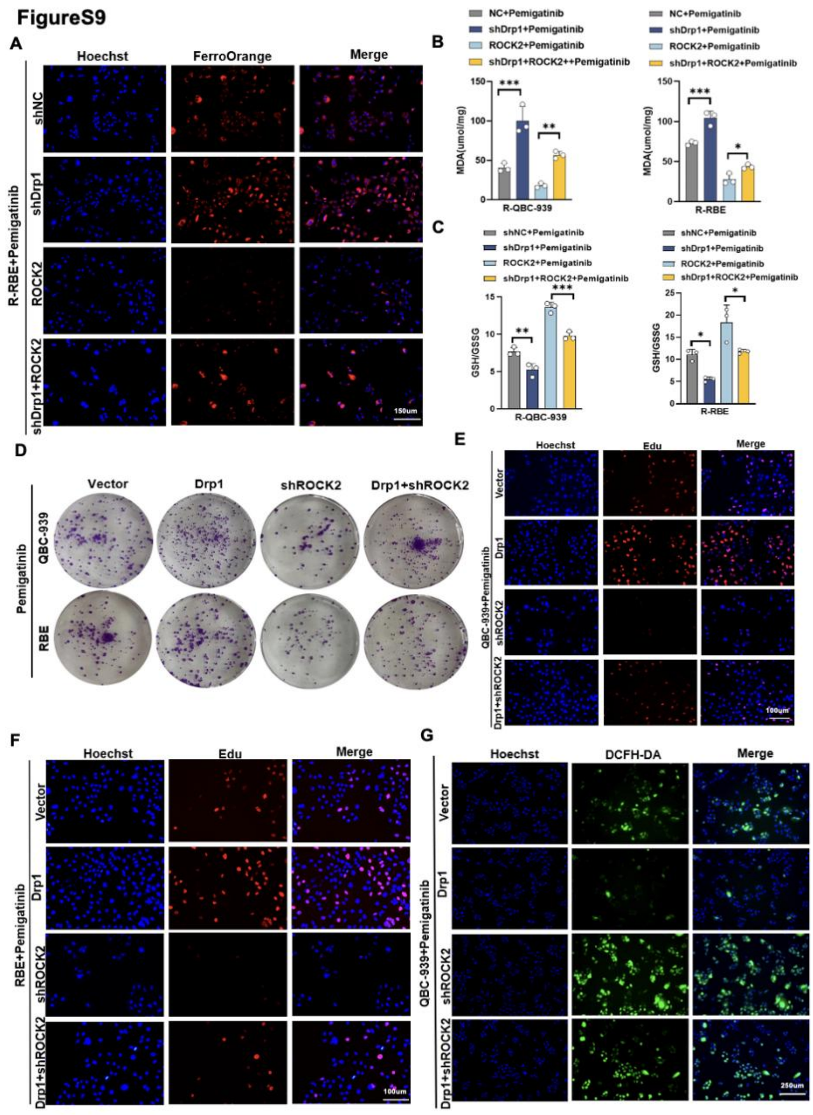

Fig.S9 ROCK2-overexpression inhibited ferroptosis and suppression of Drp1 expression blocked this ROCK2-mediated inhibition of ferroptosis

A.Intracellular ferrous ion activity was measured in R-RBE cells after treatment with Pemigatinib(5 μM, 48h at 37°C).

B.The MDA assay kit was used to determine the MDA content in the indicated groups of R-QBC-939 and R-RBE cells after treatment with Pemigatinib (5 μM, 48 h at 37 °C).Data indicated as mean ± S.D.***P < 0.001, **P < 0.01, *P < 0.05.

C. GSH/GSSG assay kit was used to determine the GSH/GSSG radio in the indicated groups of R-QBC-939 and R-RBE cells after treatment with Pemigatinib (5 μM, 48 h at 37 °C).Data indicated as mean ± S.D.***P < 0.001, **P < 0.01, *P < 0.05.

D. Cell proliferation was measured using clone formation assays in QBC-939 and RBE cells after treatment with Pemigatinib (5 μM, 48 h at 37 °C).

E-F.Cell proliferation was measured using EDU assays in QBC-939 and RBE cells after treatment with Pemigatinib (5 μM, 48 h at 37 °C).

G.Intracellular ROS levels were measured in QBC-939 cells after treatment with Pemigatinib(5 μM, 48h at 37°C).

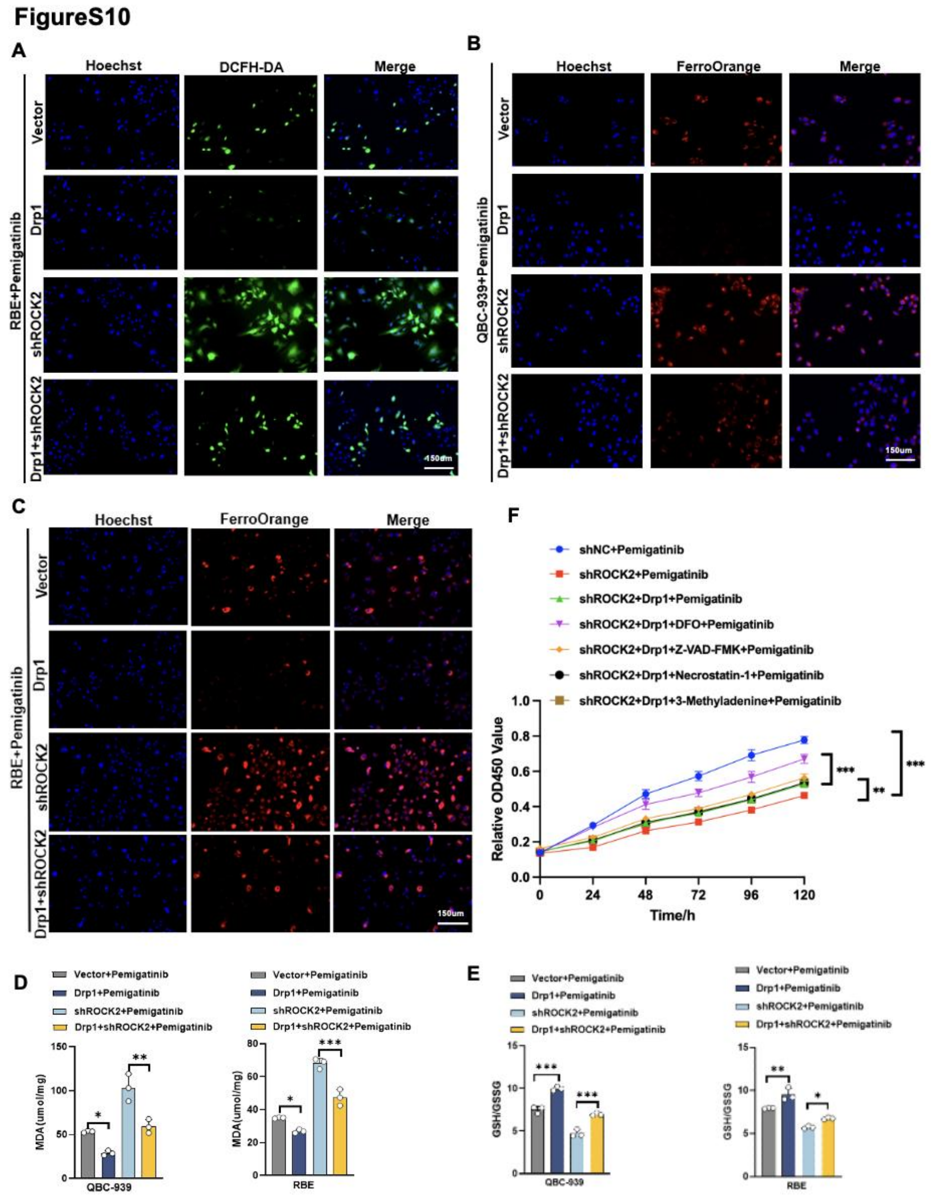

Fig.S10 ROCK2-knockdown promoted ferroptosis and Drp1-overexpression blocked this process

A.Intracellular ROS levels were measured in R-RBE cells after treatment with Pemigatinib(5 μM, 48h at 37°C).

B-C. Cell proliferation was measured using EDU assays in indicated groups in QBC-939 and RBE cells after treatment with Pemigatinib(5 μM, 48h at 37°C).

D.MDA assay kit was used to determine the MDA content in indicated groups in QBC-939 and RBE cells after treatment with Pemigatinib(5 μM, 48h at 37°C).Data indicated as mean ± S.D.***P < 0.001, **P < 0.01, *P < 0.05.

E. GSH/GSSG assay kit was used to determine the GSH/GSSG content in indicated groups in QBC-939 and RBE cells after treatment with Pemigatinib(5 μM, 48h at 37°C).Data indicated as mean ± S.D.***P < 0.001, **P < 0.01, *P < 0.05.

F. Cell viability was detected using the CCK-8 assay in indicated groups in R-RBE cells.Data indicated as mean ± S.D.***P < 0.001, **P < 0.01.

FigureS11

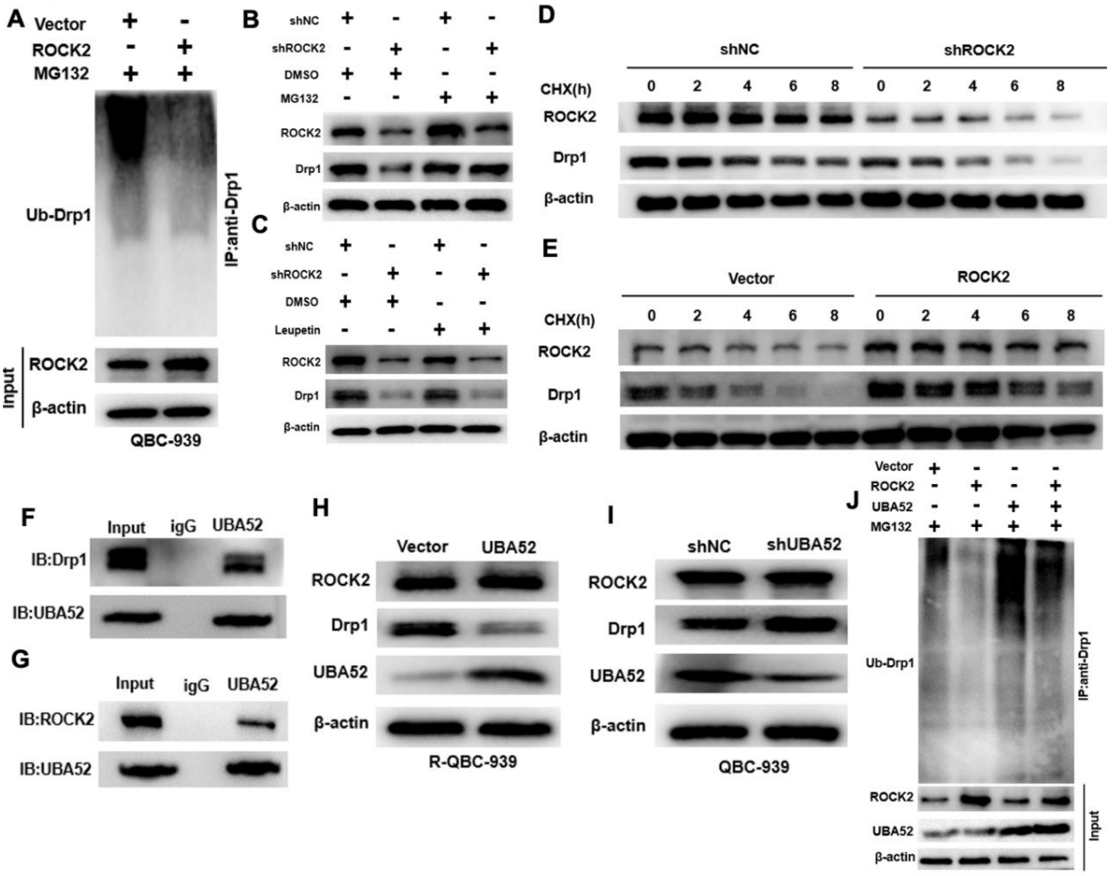

Fig.S11 ROCK2 stabilises the expression of Drp1 by competitively binding with UBA52

A. Western blot analysis was performed to detect Drp1 ubiquitination in control and ROCK2-overexpressing QBC-939 cells treated with MG132 (5 μM, 12 h at 37 °C)

B-C.Western blot analyses were performed to detect the expression levels of the indicated proteins in control and ROCK2-knockdown R-QBC-939 cells treated with MG132 (5 μM, 12 h at 37 °C) or Leupeptin (5 μM, 12 h at 37 °C).

D-E. Cells were exposed to CHX (15 μM) for the indicated time, and the degradation of ROCK2 and Drp1 was detected in the indicated groups.

F-G. Co-IP analysis of ROCK2-UBA52 and Drp1-UBA52 interactions in QBC-939 cells.

H-I. Western blot analyses were performed to detect the expression levels of the indicated proteins in UBA52-knockdown and UBA52-overexpressing cells.

J. Western blot analysis was performed to detect Drp1 ubiquitination in the indicated groups of QBC-939 cells treated with MG132 (5 μM, 12 h at 37 °C).

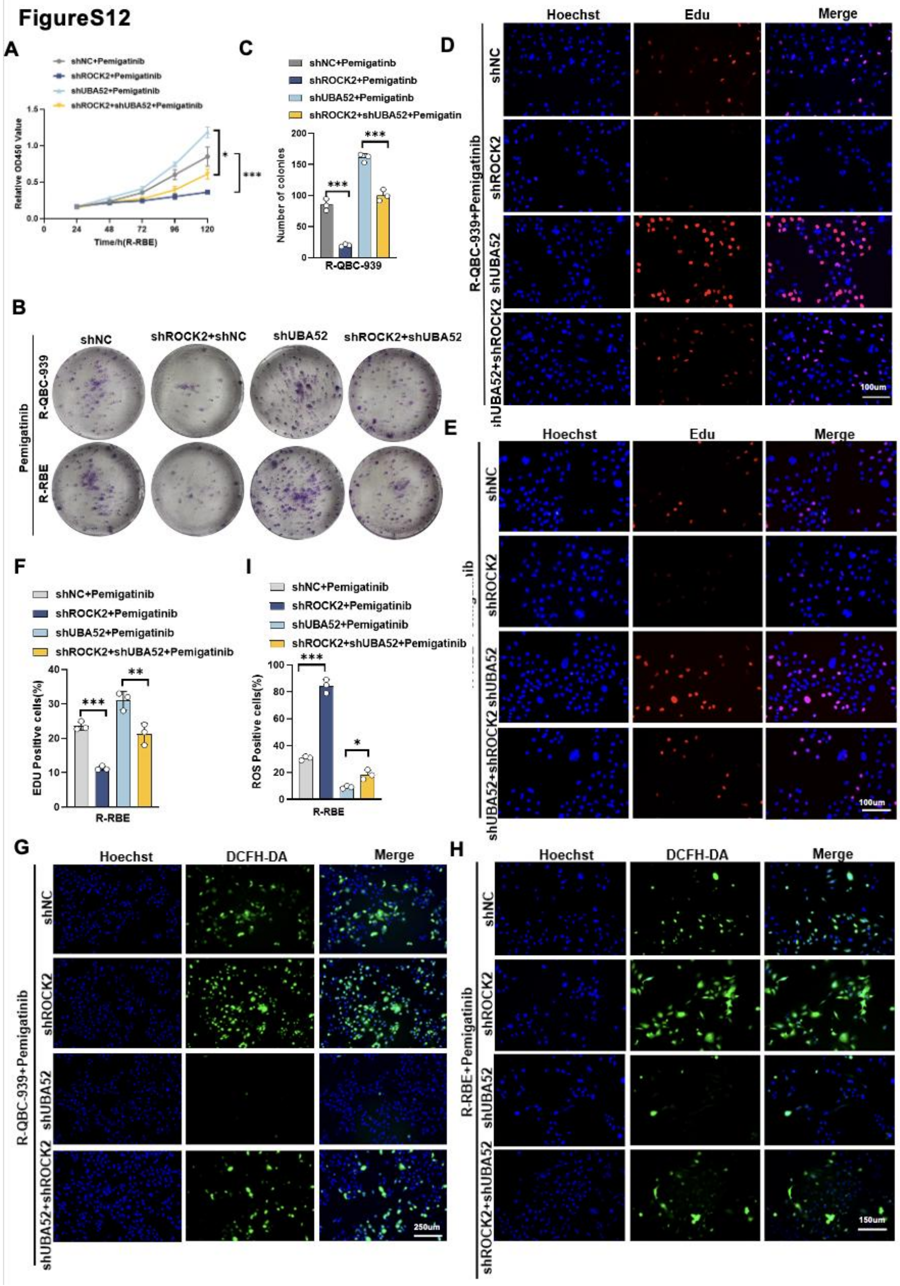

Fig.S12 ROCK2-knockdown significantly decreased CCA cell proliferation , and the effect was mitigated by the concurrent knockdown of UBA52

A. Cell viability was detected using the CCK-8 assay in indicated groups in R-RBE cells.Data indicated as mean ± S.D.***P < 0.001, *P < 0.05.

B-C. Cell proliferation was measured using clone formation assays in control and konckdown Drp1 R-QBC-939 and R-RBE cells with the indicated treatments.Data indicated as mean ± S.D.***P < 0.001.

D-F. Cell proliferation was measured EDU assays in control and konckdown Drp1 R-QBC-939 and R-RBE cells with the indicated treatments.Data indicated as mean ± S.D.***P < 0.001, **P < 0.01.

G-H. Intracellular ROS levels were measured in R-QBC-939 and R-RBE cells after treatment with Pemigatinib(5 μM, 48h at 37°C).Data indicated as mean ± S.D.***P < 0.001, *P < 0.05.

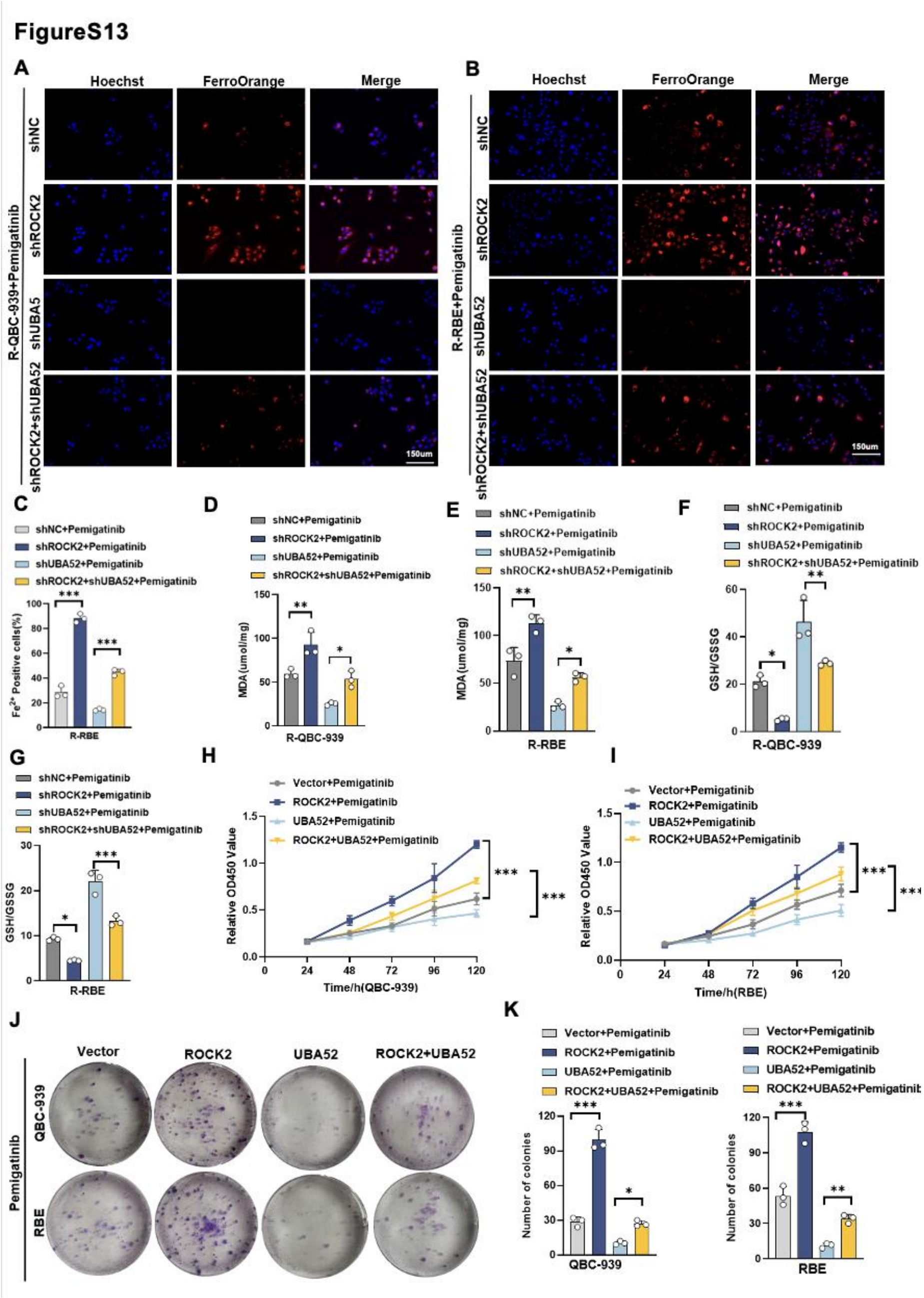

Fig.S13 ROCK2-knockdown promoted ferroptosis, and suppression of UBA52 expression blocked this ROCK2-mediated enhancement of ferroptosis

A-G.Intracellular ferrous ion activity, MDA content, and GSH/GSSG ratio were measured in the indicated groups of R-QBC-939 and R-RBE cells after treatment with Pemigatinib (5 μM, 48 h at 37 °C).Data indicated as mean ± S.D.***P < 0.001,**P < 0.01,*P < 0.05.

H-I. Cell viability was detected using the CCK-8 assay in indicated groups in QBC-939 and RBE cells after treatment with Pemigatinib (5 μM, 48 h at 37 °C).Data indicated as mean ± S.D.***P < 0.001.

J-K. Cell proliferation was measured using clone formation assays in indicated groups in QBC-939 and RBE cells after treatment with Pemigatinib (5 μM, 48 h at 37 °C).Data indicated as mean ± S.D.***P < 0.001, **P < 0.01, *P < 0.05.

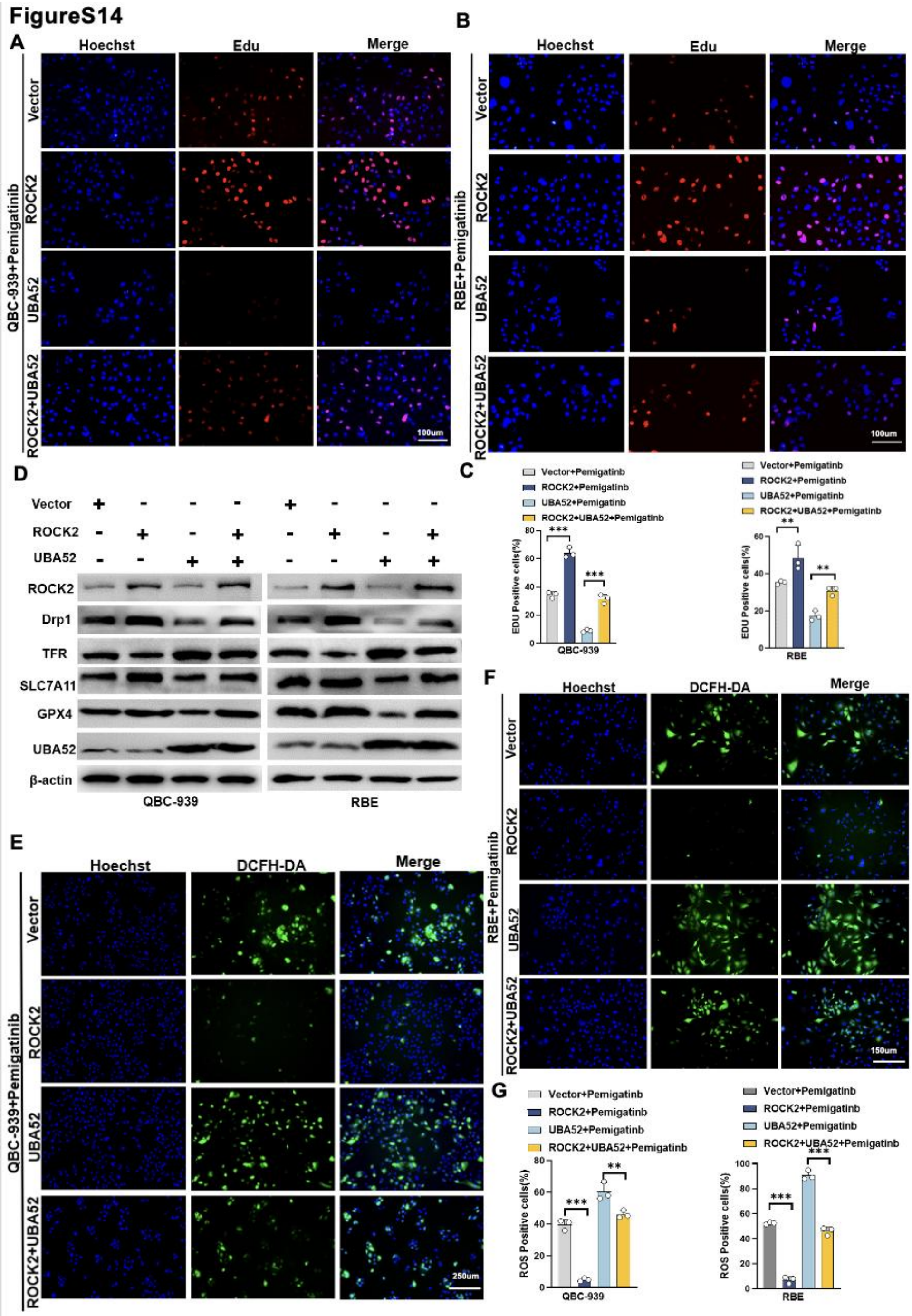

Fig.S14 ROCK2 overexpression suppresses ferroptosis, and overexpression of UBA52 blocks the weakening of ferroptosis mediated by ROCK2

A-C. Cell proliferation was measured using EDU assays in indicated groups in QBC-939 and RBE cells after treatment with Pemigatinib (5 μM, 48 h at 37 °C).Data indicated as mean ± S.D.***P < 0.001, **P < 0.01.

D. Western blot analyses were performed to detect the indicated protein expression levels in indicated groups in QBC-939 and RBE cells.

E-G. Intracellular ROS levels were measured in QBC-939 and RBE cells after treatment with Pemigatinib(5 μM, 48h at 37°C).Data indicated as mean ± S.D.***P < 0.001, **P < 0.01.

FigureS15

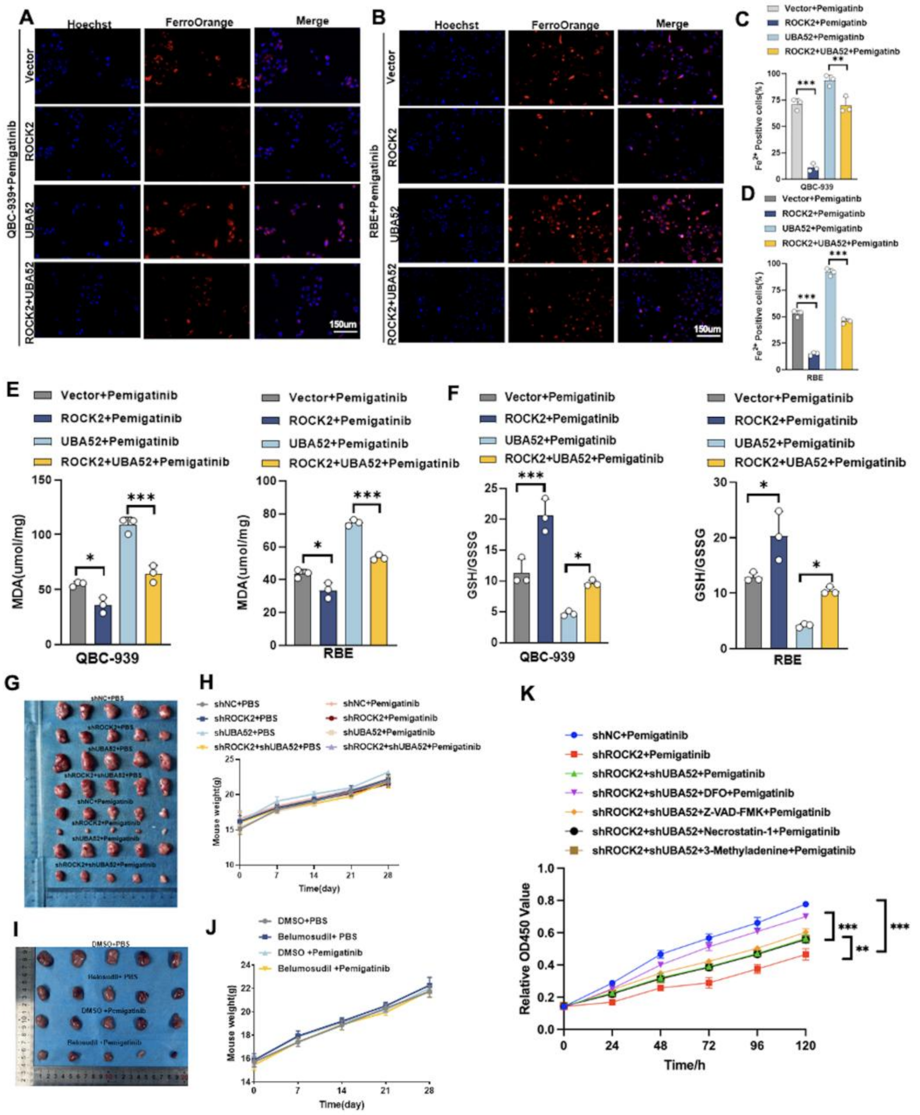

Fig.S15 ROCK2-overexpression suppression ferroptosis, and overexpression of UBA52 expression blocked this ROCK2-mediated weaken of ferroptosis *in vivo and in vitro*

A-F.Intracellular ferrous ion activity, MDA content, and GSH/GSSG ratio were measured in the indicated groups of QBC-939 and RBE cells after treatment with Pemigatinib (5 µM, 48 h at 37 °C).Data indicated as mean ± S.D.***P < 0.001, **P < 0.01, *P < 0.05

G-H. *In vivo* tumor formation was examined by subcutaneously injecting QBC-939 cells treated with the indicated protocols into the flanks of nude mice administered Pemigatinib (10 mg/kg). Representative photographs obtained 28 days after inoculation. Mice weight was measured and the corresponding Mice weight growth curves were obtained(n=5).

I-J.*In vivo* tumor formation was examined by subcutaneously injecting R-QBC-939 cells treated with Pemigatinib(10mg/kg) and Belosudil(50mg/kg) into the flanks of nude mice. Representative photographs obtained 28 days after inoculation. Mice weight was measured and the corresponding Mice weight growth curves were obtained(n=5).

K. Cell viability was detected using the CCK-8 assay in indicated groups in R-RBE cells.Data indicated as mean ± S.D.***P < 0.001, **P < 0.01.

Table S1

Reagent or resource	Source	Identifier
ROCK2	Proteintech	Cat# 21645-1-AP
Drp1	Proteintech	Cat# 12957-1-AP
TFR	Proteintech	Cat# 10084-2-AP
SLC7A11	Proteintech	Cat# 26864-1-AP
GPX4	Proteintech	Cat# 67763-1-Ig
UBA52	Zenbio	Cat# 382648
Ubiquitin	Proteintech	Cat# 10201-2-AP
GST	Proteintech	Cat#10000-0-AP
β-actin	Proteintech	Cat# 66009-1-Ig
Pemigatinib	Selleck Chemicals	Cat# 1513857-77-6
Protein A/G beads	MCE	Cat# HY-K0202
DFO(Deferasirox)	MCE	Cat# HY-17359
CHX(Cycloheximide)	MCE	Cat# HY-12320
Erastin	MCE	Cat# HY-15763
MG132	MCE	Cat# HY-13259
Leupetin	MCE	Cat# HY-18234A

Table S2

qPCR primer sequence	
ROCK2	Forward Primer:5′-GGCCGTCAGAGGAAGCTGGAGG-3′
	Reverse Primer:5′-TTAGCTTGGCTTGTTTGGAGCA-3′
Drp1	Forward Primer:5′-CTGCCTCAAATCGTCGTAGTG-3′
	Reverse Primer:5′-GAGGTCTCCGGTGACAATTC-3′
β-actin	Forward Primer:5′-CATGTACGTTGCTATCCAGGC-3′
	Reverse Primer:5′-CTCCTTAATGTCACGCACGAT-3′

Table S3

Name	Vehicle information	Target sequence
shROCK2#1	GV112	5′-GCCTTGCATATTGGTCTGGAT-3′
shROCK2#2	GV112	5′-CCTGTGTACCTGATGGAAGTT-3′
shDrp1#1	GV112	5′-CGAGATTGTGAGGTTATTGAA-3′
shDrp1#2	GV112	5′-CGGTGGTGCTAGAATTTGTTA-3′
shUBA52	GV112	5′-GCCCAGAGACACCAAAGAGTT-3'

Table S4

**Plasmid**	**Vehicle information**	**Primer sequence**
ROCK2	CV205	F:5′CTTTAAGAAGGAGATATACCATGCATCATCATCATCATCATGGAGGTG-3′
		R:5′TCGAGTGCGGCCGCAAGCTTTTAGCTAGGTTTGTTTGGGGCAAGCTG-3′
Drp1	GV350	F:5′AGGTCGACTCTAGAGGATCCCGCCACCATGGAGGCGCTAATTCCTGTC-3′
		R:5′TCCTTGTAGTCCATACCGGTCCAAAGATGAGTCTCCCGGATTTC-3′
UBA52	CV205	F:5′CTTTAAGAAGGAGATATACCATGTCCCCTATACTAGGTTATTG-3′
		R:5′TCGAGTGCGGCCGCAAGCTTTTATTTGACCTTCTTCTTGGGAC-3′
UBC(Ubiquitin-WT)	GV657	F:5′CACACTGGACTAGTGGATCCCGCCACCATGCAGATCTTCGTGAAGAC-3′
		R:5′AGTCACTTAAGCTTGGTACACCACCTCTGAGACGGAGGACCAG-3′
UBC(Ubiquitin-(K48R))	GV657	F:5′CACACTGGACTAGTGGATCCCGCCACCATGCAGATCTTCGTGAAGAC-3′
		R:5′AGTCACTTAAGCTTGGTACCGAACCACCTCTGAGACGGAGGACCAG-3′
UBC(Ubiquitin-(K63R))	GV657	F:5′CACACTGGACTAGTGGATCCCGCCACCATGCAGATCTTCGTGAAGAC-3′
		R:5′AGTCACTTAAGCTTGGTACCGAACCACCTCTGAGACGGAGGACCAG-3′
Drp1(WT)	CV205	F:5′CTTTAAGAAGGAGATATACCATGCATCATCATCATCATCATG-3′
		R:5′TCGAGTGCGGCCGCAAGCTTTCACCAAAGATGAGTCTCCCGGATTTC-3′
Drp1(K to R)	GV657	R:5′CACACTGGACTAGTGGATCCCGCCACCATGGAGGCGCTAATTCCTGTC-3′
		R:5′AGTCACTTAAGCTTGGTACCGACCAAAGATGAGTCTCCCGGATTTC-3′

Table S5

Feature		Total	ROCK2 expression		*p-value*
			Low	High	
All cases		253	118	135	-
Gender					0.548
	Man	172	78	94	
	Feman	81	48	33	
Age(years)		64(57-69)	63(55-68)	65(59-70)	***0.049***
Tumor size(cm)		2.5(1.9-3.5)	2.5(1.8-3.5)	2.5(2-3.5)	0.944
TNM stage					***0.033***
	I&II	156	81	75	
	III&IV	97	37	60	
T					***0.004***
	T1&T2	161	86	75	
	T3&T4	92	32	60	
Lymph node metastasis					0.642
	Negative	183	87	96	
	Positive	70	31	39	
Distant metastasis					0.212
	Negative	245	116	129	
	Positive	2	6	8	
Perineural invasion					0.089
	Negative	110	58	52	
	Positive	143	60	83	
